# MANF Ablation Causes Prolonged Activation of the UPR without Neurodegeneration in the Mouse Midbrain Dopamine System

**DOI:** 10.1523/ENEURO.0477-19.2019

**Published:** 2020-02-14

**Authors:** Emmi Pakarinen, Tatiana Danilova, Vootele Võikar, Piotr Chmielarz, Petteri Piepponen, Mikko Airavaara, Mart Saarma, Maria Lindahl

**Affiliations:** 1 Institute of Biotechnology, HiLIFE, University of Helsinki, Helsinki FI-00014, Finland; 2 Neuroscience Center/Laboratory Animal Center, HiLIFE, University of Helsinki, Helsinki FI-00014, Finland; 3 Department of Brain Biochemistry, Institute of Pharmacology, Polish Academy of Sciences, Kraków 31-343, Poland; 4 Division of Pharmacology and Pharmacotherapy, Faculty of Pharmacy, University of Helsinki, Helsinki FI-00014, Finland

**Keywords:** CNS, dopamine, ER stress, knock-out mice, MANF, unfolded protein response

## Abstract

Mesencephalic astrocyte-derived neurotrophic factor (MANF) is an endoplasmic reticulum (ER) localized protein that regulates ER homeostasis and unfolded protein response (UPR). The biology of endogenous MANF in the mammalian brain is unknown and therefore we studied the brain phenotype of MANF-deficient female and male mice at different ages focusing on the midbrain dopamine system and cortical neurons. We show that a lack of MANF from the brain led to the chronic activation of UPR by upregulation of the endoribonuclease activity of the inositol-requiring enzyme 1α (IRE1α) pathway. Furthermore, in the aged MANF-deficient mouse brain in addition the protein kinase-like ER kinase (PERK) and activating transcription factor 6 (ATF6) branches of the UPR pathways were activated. Neuronal loss in neurodegenerative diseases has been associated with chronic ER stress. In our mouse model, increased UPR activation did not lead to neuronal cell loss in the substantia nigra (SN), decrease of striatal dopamine or behavioral changes of MANF-deficient mice. However, cortical neurons lacking MANF were more vulnerable to chemical induction of additional ER stress *in vitro*. We conclude that embryonic neuronal deletion of MANF does not cause the loss of midbrain dopamine neurons in mice. However, endogenous MANF is needed for maintenance of neuronal ER homeostasis both *in vivo* and *in vitro*.

## Visual Abstract

**Figure:**
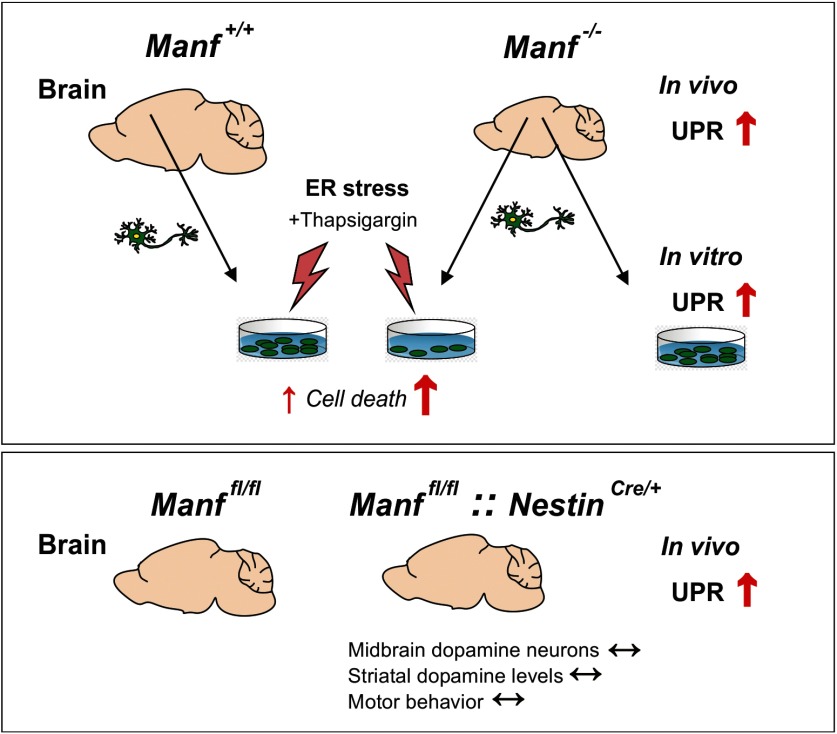

## Significance Statement

Exogenous mesencephalic astrocyte-derived neurotrophic factor (MANF) is neuroprotective in animal models of Parkinson’s disease and stroke, but the function of endogenous MANF in neurons is still elusive. This is the first study on the role of endogenous MANF in the adult mouse brain with focus on the midbrain dopamine system. We discovered chronic unfolded protein response (UPR) in the brains of mice lacking MANF. Despite activation of all three UPR pathways, we did not observe degeneration of dopamine neurons, which contrasts to the previously reported detrimental effect of chronic UPR in pancreatic β cells lacking MANF. Our findings highlight the complexity of the UPR and reveal that the terminal consequences of chronic UPR differ in cell types and context.

## Introduction

Mesencephalic astrocyte-derived neurotrophic factor (MANF) is a small, endoplasmic reticulum (ER) localized protein with survival-promoting effects and neuroprotective properties (Petrova et al., 2003; Voutilainen et al., 2009). Importantly, MANF has been associated with ER function as its expression is increased in response to several ER stress inducers and it is secreted by ER calcium depletion (Apostolou et al., 2008; Glembotski et al., 2012). ER stress is a phenomenon caused by high levels of misfolded proteins or protein aggregates. To alleviate ER stress, cells activate the unfolded protein response (UPR) involving distinct pathways initiated by three ER transmembrane stress receptors: inositol-requiring enzyme 1α (IRE1α), double-stranded RNA-activated protein kinase-like ER kinase (PERK), and activating transcription factor 6 (ATF6; Walter and Ron, 2011). These pathways enhance the degradation of misfolded proteins, the protein-folding capacity, and attenuate protein translation. If the UPR fails to restore homeostasis, sustained ER stress may trigger cell death. In fact, upregulation of UPR genes has been found in the postmortem brains of Parkinson’s disease patients and chronic ER stress is speculated to be a contributor to neurodegenerative diseases (Hoozemans et al., 2007; Hetz and Saxena, 2017; Lindholm et al., 2017). However, we lack critical studies that show the causative role of chronic ER stress and neurodegeneration.

MANF and its paralogue, cerebral dopamine neurotrophic factor (CDNF; Lindholm et al., 2007), are structurally characterized by two domains: the amino-terminal domain is predicted to bind lipids and the carboxy-terminal domain contains an ER-retention signal and is important for the anti-apoptotic activity of MANF (Parkash et al., 2009; Hellman et al., 2011; Mätlik et al., 2015). The *Manf* gene promoter carries ER stress response elements I and II, which are recognized by UPR transcription factors AFT6 and spliced X-box binding protein 1 (sXBP1), thus regulating *Manf* mRNA expression (Oh-Hashi et al., 2013; Wang et al., 2018). Fruit flies express a single CDNF/MANF homologous gene, *DmManf*, which was also shown to genetically interact with *Xbp1* (Lindström et al., 2016). MANF interacts with an ER chaperone glucose-regulated protein 78 (GRP78; Glembotski et al., 2012) and recent data show that it is the C-terminal domain of MANF that binds to the nucleotide-binding domain of GRP78, contributing to protein-folding homeostasis as a nucleotide exchange inhibitor (Yan et al., 2019).

Inactivation of the *Manf* gene in fruit flies causes defects in the formation of dopaminergic axonal bundles and decreased dopamine levels (Palgi et al., 2009). However, knock-down of neuronal *DmManf* does not affect the dopamine system in adult flies (Stratoulias and Heino, 2015). In zebrafish, knock-down of *Manf* results in a decreased number of dopamine neurons without phenotypic defects (Chen et al., 2012). In nematodes, a mutation in the *Manf* homologue was suggested to result in the gradual degeneration of dopamine neurons (Richman et al., 2018), but a recent study implicated that MANF-depleted nematodes do not after all display any dopamine phenotype (Hartman et al., 2019). We have shown that conventional MANF knock-out (KO) mice and pancreas-specific conditional KO mice develop insulin-dependent diabetes due to prolonged ER stress in pancreatic β cells, leading to loss of the β cell mass and premature death (Lindahl et al., 2014; Danilova et al., 2019a). In MANF KO mice, it was also demonstrated that MANF regulates cortical development and migration of neuronal progenitor cells (Tseng et al., 2017). Despite the suggested role of increased UPR activation behind impaired cortical development, the role of MANF in the regulation of UPR in neurons *in vivo* has remained unsolved. Whereas exogenous MANF has demonstrated protection of dopamine neurons in animal models of Parkinson’s disease (Voutilainen et al., 2009; Liu et al., 2018), the function of endogenous MANF in the dopamine neuron maintenance in mice is not known.

To study the role of endogenous MANF in midbrain dopamine neurons and in cortical neurons, we analyzed the brains of conventional and conditional MANF KO mice with the deletion of *Manf* from the neural lineage of the cells in the CNS. In this study, we aimed to characterize the survival and function of the midbrain dopamine system in conditional MANF KO mice and to examine the effect of MANF absence on ER stress and its consequences for the survival of neurons.

## Materials and Methods

### Animals

Mice were housed in an individually ventilated cage system (Mouse IVC Green Line, Tecniplast) in a specific pathogen-free facility with a 12/12 h light/dark cycle (lights on 6 A.M. to 6 P.M.) with access to food pellets (Harlan Teklad Global Diet) and water *ad libitum*. Cage enrichment was provided by bedding (aspen chips 5 × 5 × 1 mm, 4HP, Tapvei), nesting material (aspen strips, PM90L, Tapvei) and an aspen brick (100 × 20 × 20 mm, Tapvei). Development of KO mice used in the study have been previously described (Lindahl et al., 2014). Shortly, *Manf^-/-^* mice were generated from an embryonic stem cell clone MANF_D06 (EPD0162_3_D06; C57Bl/6N-Manf^tm1a(KOMP)Wtsi^). The clone included a β-galactosidase reporter cassette, which had a strong splice acceptor site between exon 2 and 3 and splicing from exon 2 to reporter cassette caused a null mutation. *Manf^fl/fl^* mice were produced by crossing *Manf^+/-^* with transgenic CAG-Flp mice following removal the β-galactosidase reporter cassette. Crossing of *Manf^fl/fl^* mice with transgenic Cre mice caused removal of exon three and splicing from exon 2 to exon 4. This resulted in a frameshift and premature stop codon and therefore with truncated *Manf* mRNA. *Manf^+/+^* and *Manf^-/-^* mice were maintained in an outbred ICR background and bred from heterozygous *Manf^+/-^* parents. *Manf^fl/fl^* mice and *Manf^fl/fl^::Nestin^Cre/+^* were in a mixed ICR/C57BL/6JRccHsd genetic background, and *Nestin^Cre/+^* mice were only in the C57BL/6JRccHsd background (Envigo). Mice used for most of the behavioral studies were bred over eight generations in the C57BL/6JRccHsd background. Only male mice were used in the behavioral testing, whereas in the other experiments the use of either female or male mice has been informed in the figure legends. Data groups of embryonic and postnatal (P1 and P14) mice include both genders. For sample collection, brains were dissected from euthanized mice, snap frozen in liquid nitrogen and stored at −75°C. For immunohistochemistry, mice were heavily anaesthetized and perfused transcardially with PBS first and then with 4% paraformaldehyde (PFA) in PBS. The experiments with mice were approved by the National Animal Experiment Board in Finland, and the project license numbers were ESAVI/10 564/04.10.07/2014 and ESAVI/11 865/04.10.07/2017.

### Blood glucose level measurements and glucose tolerance test

Blood glucose levels were measured in random-fed animals with a glucometer (Accu-Chek Aviva Glucometer, Roche Diagnostics). For the glucose tolerance test, mice were starved for 16 h in their home cages. After measurement of basal blood glucose levels, D-glucose (2 g/kg) was injected intraperitoneally, and blood glucose levels were measured at different time points during 2 h.

### Immunohistochemistry

PFA-fixed and paraffin-embedded brains were cut into 5-μm-thick sections. After paraffin removal, the sections were boiled in 10 mM citrate buffer (pH 6.0) for 10 min to retrieve antigens. The endogenous peroxidase activity was inactivated by incubation with 0.6% hydrogen peroxide in Tris-buffered saline, pH 7.4 (TBS). Sections were washed in TBS with 0.1% Tween 20 (TBS-T) and blocked with 1.5% goat/donkey serum in TBS-T. Sections were incubated with a primary antibody overnight at +4°C. Primary antibodies used were anti-MANF (1:1000, 310–100, Icosagen, RRID:AB_11135308), anti-tyrosine hydroxylase (TH; 1:1000, MAB318, Millipore, RRID:AB_2201528), anti-DAT (1:1000, MAB369, Millipore, RRID:AB_2190413), and anti-neuronal nuclear protein (anti-NeuN) NeuN (1:500, MAB377, Millipore, RRID:AB_2298772). After incubation with biotinylated secondary antibodies, sections were detected with Vector diaminobenzidine peroxidase substrate kit (Vector Laboratories). Secondary antibody used was horse anti-mouse (1:200, BA-2000, Vector Laboratories).

In immunofluorescence staining, blocking solution was added for 1 h after antigen retrieval and the primary antibody was kept overnight. The primary antibodies used were anti-TH (1:500, MAB318, Millipore, RRID:AB_2201528) and anti-GRP78 (1:500, ab21685, Abcam, RRDI:AB_2119834). Sections were incubated with fluorescent-labeled secondary antibodies, and after a 1-h incubation, they were washed three times with TBS-T and mounted with a medium containing 4′−6-diamidino-2-phenylindole (DAPI; H-1200, Vectashield, Vector Laboratories).

### Counting of dopamine neurons

To count the relative number of dopamine neurons in the substantia nigra (SN), four nigral sections within an interval of 100–120 μm were selected for the staining. TH-stained sections of the SN were scanned with an automated scanner (3DHistech). An area of the SN pars compacta (SNpc) from each hemisphere was defined separately based on morphologic landmarks and an estimation of dopamine neuron number was performed with a MATLAB algorithm based on intensity from TH-immunoreactive sections (Penttinen et al., 2016). In the paper of Penttinen et al. (2016), the method was validated by comparing stereological counting with this new method and analyzing dopamine neurons in different PD models. Counts were performed bilaterally and results are presented as relative change in the cell number per stained section.

### Optical density measurement

Images of TH-immunostained and DAT-immunostained striatal sections were scanned with a 3DHistech scanner. Striata were defined from pictures, and optical density was measured with an Image-Pro Analyzer 7.0. The optical density of a cortical region above the striatum was subtracted as a background signal. Six sections per animal were used for the analysis. The results of integrated optical density are shown as an average and percentage of the control mice.

### Measurement of dopamine and serotonin levels

The neurotransmitter levels from dissected striata were measured by high-performance liquid chromatography (HPLC) as previously described (Airavaara et al., 2006). Tissue samples were homogenized in a solution containing 0.2 M HClO_4_ and antioxidants following centrifugation at 14,000 rpm for 35 min. Supernatant was further filtered with Vivaspin columns by centrifugation at 9000 rpm for 35 min. From striatal tissues samples, concentrations of dopamine, 3,4-dihydroxyphenylacetic acid (DOPAC), homovanillic acid (HVA), serotonin, and 5-hydroxyindoleacetic acid were analyzed with a HPLC including an electrochemical detector.

### Real-time quantitative PCR (qPCR)

Total RNA was isolated by phenol–chloroform extraction with TRI Reagent (Molecular Research Center). Briefly, TRI reagent (Invitrogen, Thermo Fisher Scientific) was added to samples, followed by mechanical disruption by grinding with a pestle or by pulling through a needle by a syringe. Chloroform (1:4) was added to the samples, which were centrifuged at 13,000 rpm for 15 min. The aqueous phase was collected and isopropanol (1:1) added. For small samples, glycogen (Thermo Fisher Scientific) was added to visualize the pellet. After incubation, the samples were centrifuged at 13,000 rpm for 15 min, followed by washing of the pellet twice with 75% ethanol. The pellet was air-dried and dissolved in water. RNA concentration was measured by NanoDrop and equal amounts of RNA were used for synthesizing complementary DNA. RNA quality was assessed by measuring UV absorbances of the RNA samples. Reverse transcription reaction was catalyzed by RevertAid Premium Reverse Transcriptase (Thermo Fisher Scientific) or Maxima H Minus Reverse Transcriptase (Thermo Fisher Scientific) in the presence of 10 mM NTP mix (Fermentas UAB) and oligo-d(T) (Metabion International). qPCR was performed with Lightcycler 480 SYBR Green I Master mix (Roche Diagnostics) using Lightcycler 480 Real-Time PCR System (Roche). The mRNA levels of the target gene were normalized to levels of β-actin as a reference gene, and quantification was performed by a standard curve method. Primer sequences were designed to span exon-exon junctions. Primer sequences were: *Atf4* F 5′-ATG GCC GGC TAT GGA TGA T-3′, *Atf4* R 5′-CGA AGT CAA ACT CTT TCA GAT CCA TT-3′, *Atf6α* F 5′-GGA CGA GGT GGT GTC AGA G-3′, *Atf6α* R 5′-GAC AGC TCT TCG CTT TGG AC-3′, *Bcl10* F 5′-AAA CTG GAG CAC CTC AAA GG-3′, *Bcl10* R 5′-TCT CAT CGG AAT TGC ACC TA-3′, *Bdnf* F 5′-TAC CTG GAT GCC GCA AAC AT-3′, *Bdnf* R 5′-GCT GTG ACC CAC TCG CTA AT-3′, *Cdnf* F 5′-GCT CAG ATG CCA AAG GAA AA-3′, *Cdnf* R 5′-TAG GAT CTT GGT GGC TGC AT-3′, *Chop* F 5′-CCA ACA GAG GTC ACA CGC AC-3′, *Chop* R 5′-TGA CTG GAA TCT GGA GAG CGA-3′, *Dat* F 5′*-*AAC CTG TAC TGG CGG CTA TG-3′, *Dat* R 5′-GCT GAC CAC GAC CAC ATA CA-3′, *Grp78* F 5′-ACC CTT ACT CGG GCC AAA TT-3′, *Grp78* R 5′-AGA GCG GAA CAG GTC CAT GT-3′, *Manf* F 5′-GAC AGC CAG ATC TGT GAA CTA AAA-3′, *Manf* R 5′-TTT CAC CCG GAG CTT CTT C-3′, *sXbp1* F 5′-GAG TCC GCA GCA GGT G-3′, *sXbp1* R 5′-GTG TCA GAG TCC ATG GGA-3′, *Th* F 5′-CCC AAG GGC TTC AGA AGA G-3′, *Th* R 5′-GGG CAT CCT CGA TGA GAC T-3′, *tXbp1* F 5′-CAC CTT CTT GCC TGC TGG AC-3′, *tXbp1* R 5′-GGG AGC CCT CAT ATC CAC AGT-3′, *Txnip* F 5′-TCA AGG GCC CCT GGG AAC ATC-3′, *Txnip* R 5′-GAC ACT GGT GCC ATT AAG TCA G-3′.

### Western blotting

Samples were homogenized in lysis buffer composed of 10 mM Tris-HCl (pH 8.0), 300 mM NaCl, 4 mM EDTA, 0.2% Triton X-100, and a protease inhibitor cocktail (cOmplete, Roche, 04693159001). Also, a phosphatase inhibitor (PhosStop, Roche, 04906837001) or 1 mM sodium orthovanadate was added to inhibit phosphatase activity. Homogenates were incubated on ice for 1 h, followed by centrifugation at 13,000 rpm for 20 min. The supernatant was collected, and protein concentrations were measured with DC protein assay (Bio-Rad Laboratories) according to the manufacturer’s instructions. To equalize protein concentrations, samples were diluted in lysis buffer and then mixed with Laemmli buffer containing 2% 2-mercaptoethanol. Samples were heated at +95°C for 5 min. For DAT detection, striatal samples were homogenized in a 10 mM HEPES (pH 7.2), buffer containing 0.3 M sucrose, 1 mM EDTA and protease inhibitors. The samples were incubated with Laemmli buffer at +37°C for 50 min and run on self-made or pre-cast gels (Bio-Rad). Proteins were transferred onto nitrocellulose membranes, which were blocked with 5% milk or 5% BSA in TBS-T for 1 h and incubated with primary antibody solution overnight. The primary antibodies used were: anti-GRP78 (1:1000, sc-1051, Santa Cruz Biotechnology, RRDI:AB_2119994), anti-eIF2α (1:1000, 9722, Cell Signaling, RRID:AB_2230924), anti-peIF2α (1:1000, 9721, Cell Signaling, RRID:AB_330951), anti-GAPDH (1:3000, MAB374, Millipore, RRID:AB_2107445), anti-TH (1:1000, MAB318, Millipore, RRID:AB_2201528), anti-DAT (1:1000, MAB369, Millipore, RRID:AB_2190413), anti-p-NF-κB (1:1000, 3033, Cell Signaling, RRID:AB_10859369), and anti-NF-κB (1:1000, 8242, Cell Signaling, RRID:AB_331284). Secondary antibody was applied for 1 h and detected with chemiluminescence ECL Blotting Substrate (Pierce, Thermo Fisher Scientific). The secondary antibodies used were: goat anti-mouse (1:3000, P0447, Dako), donkey anti-rabbit (1:3000, NA9340V, GE Healthcare), goat anti-rat (1:1000), and rabbit anti-goat (1:1500, P0449, Dako). Protein bands were quantified with ImageJ software. Protein amounts were normalized to GAPDH.

### Primary dopamine cell cultures

To culture midbrain dopamine neurons from single embryos, the method of micro-island cultures was used. Micro-islets were drilled into four-well plates and coated with poly-ornithine. Midbrain floors were dissected from E13.5 embryos, collected in Dulbecco’s media and washed with HBSS. Cells were detached by trypsin treatment at +37°C for 20–30 min. To stop the reaction, fetal bovine serum (FBS) was added and cells were triturated. After the sedimentation of cells, the supernatant was collected and the trituration repeated. Cells were centrifuged for 5 min at 1000 rpm. The pellet was washed twice with the media and dissolved in the media. Approximately 2000–3000 cells were added to each micro-island. Some plates were fixed on the first day *in vitro* (DIV1). For other plates, half of the media was changed at DIV2 and fixed at DIV5. Cells were fixed with 4% PFA, washed and permeabilized with 0.2% Triton X-100 in PBS. Samples were blocked with 5% horse serum + 0.2% Triton X-100 in PBS and incubated with anti-TH antibody (1:1000, MAB318) overnight. Specimens were incubated with secondary antibody (1:400, Alexa Fluor 488 Goat Anti-mouse, Life Technologies, A11029) for 1 h, and nuclei were stained with DAPI (1:5000) for 10 min. Images from micro-islands were taken with a Leica fluorescent camera DC300F and TH+ cells were quantified from images with ImageJ (Rueden et al., 2017). Each data point was an average of at least two micro-islands.

### Primary cortical cell cultures

Cortex was collected from mouse fetuses at gestational day 16 (E16) or E17 separately and later genotyped. Cortical pieces were washed twice with HBSS. Samples were incubated with 0.5% trypsin for 15 min at +37°C, followed by addition of HBSS + 10% FBS and DNase I. Centrifuged pellets were washed twice with HBSS+FBS solution. Neurobasal medium (Invitrogen) supplemented with B27 (1×, Invitrogen), 500 μM glutamine, and primocin was added on top of the pellet and pipetted until tissue pieces were separated. Cells were centrifuged and washed with media. Dissociated neurons were counted, and cells were plated on poly-ornithine coated 96-well or 12-well plates at the amount per well of 2.5 × 10^4^ and 4 × 10^5^, respectively. Half of the medium was changed every 2–4 d. Cortical neurons cultured on 12-well plates were treated with 20 μM 5-fluoro-2′-deoxyuridine (F0503, Sigma-Aldrich) and 20 μM uridine (U3750, Sigma-Aldrich) from DIV2 to DIV5 to reduce the number of glial cells. Treatments were performed during media change at DIV9. Neurons cultured on 96-well plates were fixed with 4% PFA. They were permeabilized with 0.2% Triton X-100 in PBS for 15 min and blocked with 5% horse serum in 0.2% Triton X-100 in PBS. A primary antibody (anti-NeuN, 1:500, MAB377, Millipore) was added to the blocking medium and incubated overnight. After incubation with a secondary antibody (1:400, Alexa Fluor 488 goat anti-mouse, Life Technologies, A11029) for 1 h, the nuclei were stained with DAPI. Cultures were imaged with a CellInsight CX5 instrument (Thermo Fisher Scientific) and the number of NeuN-positive cells was analyzed automatically by designed image analysis workflow in CellProfiler and CellProfiler Analyst programs (Carpenter et al., 2006; Jones et al., 2009). Neurons grown on 12-well plates were used for RNA extraction or Western blotting in the same way as described above.

### Multiple static rods

The performance of a mouse on five rods with increasing diameter (rod 1: 27 mm, rod 2: 21 mm, rod 3: 15 mm, rod 4: 11 mm, rod 5: 8 mm) was measured. At the beginning of a test, a mouse was placed at the end of the round beam facing away from the supporting platform. The latencies to turn 180°, walk 60 cm, and possible fall off were measured within a maximum of 2 min.

### Wire hanger test

A mouse was placed in the middle of the horizontal hanger grid. The mouse was observed for 1 min, and the latency to fall was measured.

### Accelerating rotarod

Mice were trained once on an accelerating rotarod (from 4 to 40 rpm during 4 min). Later on, the same day, the latency to fall was measured with the 4-min cutoff time.

### Open-field activity

Mice were placed in the corner of an open-field arena (30 × 30 cm, Med Associates). The horizontal activity was monitored for 30 min. For testing amphetamine-induced locomotor activity, mice were first habituated in the open-field arenas for 30 min. Mice were then injected with 3 mg/kg D-amphetamine intraperitoneally and placed back in the same chambers. The horizontal activity was measured for another 90 min.

### Barnes maze

A circular elevated platform (diameter 100 cm) contained 20 equally distributed holes (diameter, 5 cm) around the perimeter. An escape box was placed under one of the holes. Mice were trained to find the escape box during training sessions of a maximum of 3 min per time. The trial was ended either when the animal entered the box or 3 min had elapsed; in the latter case the animal was guided to the box by hand and allowed to enter it. Training was conducted three times per day (with an inter-trial interval of at least 1 h) on three consecutive days. On day 4, for testing of memory in probe trial 1, the box was removed and mice had 1.5 min to navigate to the right place. For reverse training, the escape box was set at the opposite side of the maze. Reverse trainings of 3 min occurred three times per day on two consecutive days. The maze was cleaned with water between every animal. The trials were recorded with video tracking software Ethovision XT10 (Noldus). The distance traveled and the latency to find the escape box were measured during the training trial; in addition, the time spent in a zone surrounding the target hole was measured in the probe trial.

### Statistical analysis

Statistical analysis was performed using either SPSS Statistics (version 24, IBM) or GraphPad Prism (version 7.04, GraphPad Software). Student’s *t* test was used when comparing two groups when data were normally distributed. If data were not normally distributed or Levene’s test for equality of variances showed unequal variances, the Mann–Whitney *U* test was used to compare two groups. If only part of the large dataset was not normally distributed, the nonparametric Mann–Whitney *U* test was still used to make consistent analysis. Data with three groups and one factor were analyzed with one-way ANOVA, followed by Tukey’s *post hoc* test, and if data were not normally distributed, the nonparametric Kruskal–Wallis test followed by Mann–Whitney *U* test was used. For tests with many measurements from the same subject, we used two-way ANOVA for repeated measures. For the data, where thapsigargin was used as a treatment, two-way ANOVA was used to analyze genotype–treatment interaction, and it was followed by Tukey’s *post hoc* test. The behavioral tests and analysis were performed blinded. All results are presented as mean ± SEM and regarded as significant when *p *<* *0.05. The statistical test used are listed in Table 1 and Extended Data Table 1-1, but significant exact *p* values from Mann–Whitney *U* test and Tukey’s *post hoc* test are presented in the figures due to clarity.

**Table 1.** Statistical analysis

Dataset	Data structure	Type of test	Power
Figure 1*A*	Non-normal distribution	Mann–Whitney *U*	Significant *p* values in the figure
Figure 1*B*	Non-normal distribution	Kruskal–Wallis, Mann–Whitney *U*	*H*_(2)_ = 11.33, *p* = 0.003
Figure 1*D*,*E*	Non-normal distribution	Mann–Whitney *U*	No significance
Figure 1*F*	Normal distribution	One-way ANOVA, Tukey's HSD	4-month-old mice *F*_(2,22)_ = 20.42, *p* < 0.001; 12-month-old mice *F*_(2,22)_ = 6.35, *p* = 0.007
Figure 1*G*	Normal distribution	Two-tailed *t* test and one-way ANOVA	2-month-old mice *t*_(30)_ = 0.93, *p* = 0.360; 16-month-old mice *F*_(2,22)_ = 0.43, *p* = 0.659
Figure 1*H*	Normal distribution	Two-way RM ANOVA	Genotype × time interaction *F*_(6,54)_ = 0.88, *p* = 0.517
Figure 2*A*–*G*	Non-normal distribution	Mann–Whitney *U*	Significant *p* values presented in the figure
Figure 2*H*	Normal distribution	Two-tailed *t* test	Cortex *Txnip t*_(6)_ = 1.046, *p* = 0.34; Cortex *Bcl10 t*_(12)_ = 0.59, *p* = 0.564; SN *Txnip t*_(10)_ = 1.15, *p* = 0.279; SN *Bcl10 t*_(10)_ = 0.41, *p* = 0.688
Figure 3*A*	Normal distribution	One-way ANOVA, Tukey's HSD	*Atf6α F*_(2,13)_ = 0.08, *p* = 0.924; *Atf4 F*_(2,13)_ = 0.08, *p* = 0.927; *Chop F*_(2,13)_ = 1.19, *p* = 0.334; *Grp78 F*_(2,13)_ = 9.70, *p* = 0.003; *sXbp1 F*_(2,13)_ = 99.98, *p* < 0.001; *tXbp1 F*_(2,13)_ = 0.032, *p* = 0.968
Figure 3*B*	Normal distribution	One-way ANOVA, Tukey's HSD	*Atf6α F*_(2,11)_ = 5.88, *p* = 0.018; *Atf4 F*_(2,11)_ = 7.13, *p* = 0.010; *Chop F*_(2,11)_ = 0.25, *p* = 0.781; *Grp78 F*_(2,11)_ = 15.18, *p* = 0.001; *sXbp1 F*_(2,10)_ = 134.32, *p* < 0.001; *tXbp1 F*_(2,11)_ = 5.39, *p* = 0.023
Figure 3*C*	Normal distribution	One-way ANOVA	*Txnip F*_(2,11)_ = 1.22, *p* = 0.331; *Bcl10 F*_(2,11)_ = 1.13, *p* = 0.358
Figure 4*A*	Normal distribution	Two-tailed *t* test	*t*_(8)_ = 1.89, *p* = 0.095
Figure 4*C*	Normal distribution	Two-tailed *t* test	TH-fibers *t*_(8)_ = 0.32, *p* = 0.760; DAT-fibers *t*_(8)_ = 0.71, *p* = 0.499
Figure 4*E*	Non-normal distribution	Kruskal–Wallis, Mann–Whitney *U*	DA *H*_(2)_ = 6.68, *p* = 0.035, DOPAC *H*_(2)_ = 6.68, *p* = 0.541; HVA *H*_(2)_ = 0.422, *p* = 0.810; 5-HT *H*_(2)_ = 1.618, *p* = 0.445; 5-HIAA *H*_(2)_ = 0.23, *p* = 0.893
Figure 5*A*	Non-normal distribution	Kruskal–Wallis, Mann–Whitney U	Time to turn on the rod 1 *H*_(2)_ = 9.60, *p* < 0.001
Figure 5*B*	Non-normal distribution	Kruskal–Wallis	*H*_(2)_ = 3.07, *p* = 0.216
Figure 5*C*	Normal distribution	One-way ANOVA	*F*_(2,25)_ = 2.77, *p* = 0.082
Figure 5*D*	Normal distribution	One-way ANOVA	*F*_(2,22)_ = 1.44, *p* = 0.259
Figure 5*E*	Normal distribution	One-way ANOVA, Tukey's HSD	*F*_(2,25)_ = 3.99, *p* = 0.031
Figure 5*F*	Normal distribution	One-way ANOVA	*F*_(2,19)_ = 1.94, *p* = 0.172
Figure 5*G*	Normal distribution	Two-way RM ANOVA	Genotype × time interaction *F*_(46,529)_ = 1.10, *p* = 0.314
Figure 5*H*	Normal distribution	Two-way RM ANOVA	Genotype × time interaction *F*_(46,460)_ = 0.65, *p* = 0.964
Figure 5*I*	Normal distribution	Two-way RM ANOVA	Trials 1–9 *F*_(16,176)_ = 0.57, *p* = 0.905, trials 10–15 *F*_(10,110)_ = 0.39, *p* = 0.947
Figure 5*J*	Non-normal distribution	Kruskal–Wallis	Trial I *H*_(2)_ = 0.044 *p* = 0.978, Trial II *H*_(2)_ = 0.244 *p* = 0.885
Figure 6*A*	Normal distribution	Two-tailed *t* test	*t*_(13)_ = 1.59, *p* = 0.137
Figure 6*B*	Normal distribution	One-way ANOVA, Tukey's HSD	*Atf6α F*_(2,12)_ = 3.42, *p* = 0.067; *Atf4 F*_(2,12)_ = 7.76 *p* = 0.007; *Chop F*_(2,12)_ = 3.72, *p* = 0.056 ; *Grp78 F*_(2,12)_ = 13.05, *p* = 0.001; *sXbp1 F*_(2,12)_ = 17.85, *p* = 0.003; *tXbp1 F*_(2,12)_ = 19.42, *p* = 0.002; *Bcl10 F*_(2,14)_ = 1.84, *p* = 0.196
Figure 6*C*	Normal distribution	Two-way ANOVA, Tukey's multiple comparison	Genotype × treatment interaction *F*_(4,36)_ = 2.82, *p* = 0.039
Figure 6*D*–*K*	Normal distribution	Two-way ANOVA, Tukey's multiple comparison	Genotype × treatment interactions: *Atf6α F*_(2,20)_ = 0.40, *p* = 0.677; *Atf4 F*_(2,20)_ = 1.40, *p* = 0.270; *Chop F*_(2,20)_ = 0.07, *p* = 0.934; *Grp78 F*_(2,20)_ = 0.71, *p* = 0.504; *sXbp1 F*_(2,18)_ = 5.73, *p* = 0.012; *tXbp1 F*_(2,20)_ = 0.19, *p* = 0.826; *Bcl10 F*_(2,20)_ = 1.72, *p* = 0.203, *Txnip F*_(2,16)_ = 1.40, *p* = 0.275
Figure 6*M*	Normal distribution	Two-way ANOVA	Genotype × treatment interactions: p-NF-κB/NF-κB *F*_(1,14)_ = 1.698, *p* = 0.214

RM, repeated measures; HSD, honestly significant difference. See Extended Data Table 1-1 for statistical analysis of the Extended Data.

**Figure 1.**
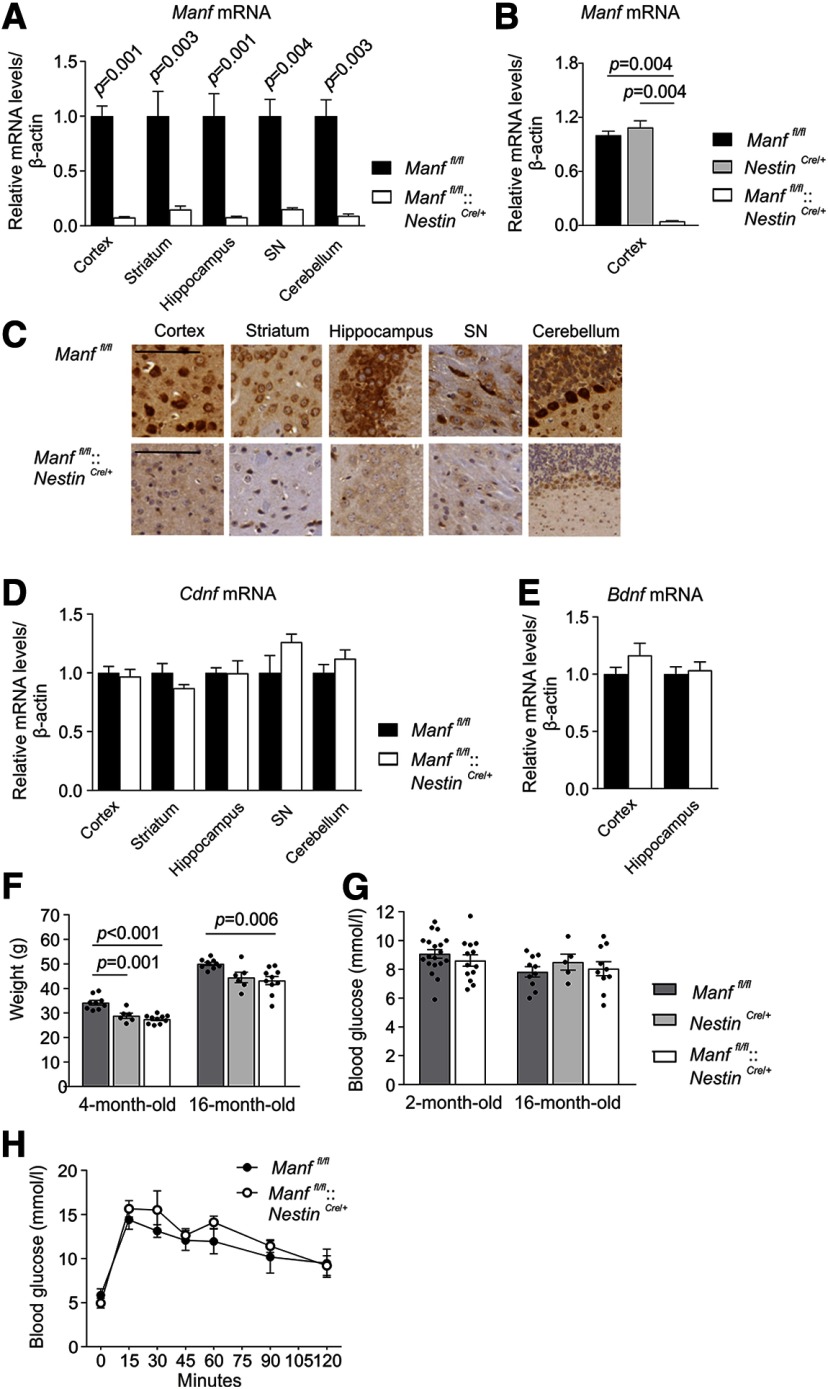
Generation of CNS-specific MANF KO mice. ***A***, *Manf* mRNA levels in different brain areas of two-month-old *Manf^fl/fl^* and *Manf^fl/fl^::Nestin^Cre/+^* female mice determined by qPCR (*n = *4–9). Results are scaled to the average value of the control samples. ***B***, *Manf* mRNA levels of 16-month-old *Manf^fl/fl^*, *Manf^fl/fl^::Nestin^Cre/+^*, and *Nestin^Cre/+^* male mice (*n *=* *5–6). ***C***, Immunoreactivity for endogenous MANF with hematoxylin counterstaining in the brains of *Manf^fl/fl^* and *Manf^fl/fl^::Nestin^Cre/+^* mice. Scale bar: 200 μm. ***D***, *Cdnf* mRNA levels in different brain areas of two-month-old female mice (*n = *4–9). ***E***, *Bdnf* mRNA levels in the cortex and hippocampus of two-month-old female mice (*n *=* *6–8). ***F***, Weights measured from *Manf^fl/fl^*, *Manf^fl/fl^::Nestin^Cre/+^*, and *Nestin^Cre/+^* male mice at the age of four and 16 months (*n = *6–14). ***G***, Blood glucose levels of *Manf^fl/fl^::Nestin^Cre/+^* female and male mice and their littermate controls (*n = *3–10). ***H***, Glucose tolerance test performed for 16-month-old *Manf^fl/fl^* and *Manf^fl/fl^::Nestin^Cre/+^* female mice (*n *=* *5). Mann–Whitney *U* test, Student’s *t* test, one-way and two-way ANOVA followed by Tukey’s *post hoc* test, or Kruskal–Wallis followed by Mann–Whitney *U* test were used for statistical analysis. Data are presented as mean ± SEM.

## Results

### Conditional deletion of MANF in the nervous system

Conditional MANF KO mice were produced by deleting an exon three of the *Manf* gene by crossing the floxed mice (*Manf^fl/fl^*) with transgenic *Nestin^Cre/+^* mice. Cre recombinase expressed under the Nestin promoter in *Nestin^Cre/+^* transgenic mice is induced predominantly in the CNS (Tronche et al., 1999). Later, it was reported that in the *Nestin^Cre/+^* line used, recombination is observed in neural stem cells and neural progenitor cells from E12.5 but with incomplete efficiency (Dubois et al., 2006). Since MANF is mainly expressed in neurons in the adult mouse brain (Lindholm et al., 2008; Tseng et al., 2017; Danilova et al., 2019a,b), this recombination is expected to remove most of the MANF in the brain. Consequently, qPCR analysis showed barely detectable levels of *Manf* mRNA analyzed from the brain lysates from different brain areas of *Manf^fl/fl^::Nestin^Cre/+^* mice compared with littermate *Manf^fl/fl^* mice (Fig. 1*A*; Table 1). Brain regions analyzed were selected based on their normally high MANF expression (Lindholm et al., 2008). Importantly, *Manf* mRNA levels were similar in the cortex between control *Manf^fl/fl^* and *Nestin^Cre/+^* mice, and reduced as expected in *Manf^fl/fl^::Nestin^Cre/+^* mice (Fig. 1*B*; Table 1). This result indicates the comparable levels of *Manf* mRNA in *Manf^fl/fl^* and *Nestin^Cre/+^* mice. Consistent with the *Manf* mRNA levels, immunoreactivity for MANF with a validated MANF-specific antibody was detected in the brains of *Manf^fl/fl^* mice, but the signal was almost fully lost in *Manf^fl/fl^::Nestin^Cre/+^* mouse brain counterstained with hematoxylin (Fig. 1*C*). The loss of MANF did not affect *Cdnf* mRNA expression levels in the *Manf^fl/fl^::Nestin^Cre/+^* brain areas (Fig. 1*D*; Table 1) or the *Bdnf* mRNA levels measured from the cortical and hippocampal tissue (Fig. 1*E*; Table 1).

In contrast to global *Manf^-/-^* mice in the ICR strain that die prematurely (Lindahl et al., 2014) and *Manf^-/-^* mice in the C57BL/6 strain that are perinatal lethal (Neves et al., 2016; Bell et al., 2019), *Manf^fl/fl^::Nestin^Cre/+^* mice were viable and visibly similar to littermates when followed until the age of 18 months. The body weights of *Manf^fl/fl^::Nestin^Cre/+^* mice were reduced at four and 16 months of age compared with the *Manf^fl/f^* ^l^ controls but when comparing *Manf^fl/fl^::Nestin^Cre/+^* mice with *Nestin^Cre/+^* mice, there was no difference in body weight (Fig. 1*F*; Table 1). Since conventional MANF KO mice develop diabetes by the age of seven weeks (Lindahl et al., 2014), we followed the blood glucose levels to rule out the possible development of hyperglycemia. In line with the previously published data (Lindahl et al., 2014), blood glucose levels of random-fed *Manf^fl/fl^::Nestin^Cre/+^* mice stayed similar to *Manf^fl/fl^* controls until the age of 16 months (Fig. 1*G*; Table 1). The glucose tolerance test measuring insulin secretion and sensitivity was performed by injecting glucose followed by measuring the rate of glucose clearance from the blood stream. Both *Manf^fl/fl^* and *Manf^fl/fl^::Nestin^Cre/+^* mice showed similar glucose clearance over time (Fig. 1*H*; Table 1).

### MANF deficiency causes robust activation of IRE1α signaling pathway in the brain

Previously it has been shown that loss of MANF in pancreatic β cells leads to chronic UPR activation *in vivo* (Lindahl et al., 2014). Recently, it was also demonstrated that ablation of MANF in cartilage results in upregulation of ER chaperones *in vivo* (Bell et al., 2019). Therefore, we examined the expression of UPR-related genes in the brains of both conventional *Manf^-/-^* and conditional *Manf^fl/fl^::Nestin^Cre/+^* KO mice. The studied UPR-related genes were selected from all three UPR branches in addition to the major UPR sensor GRP78 (alias BiP), which in normal conditions binds to these ER transmembrane UPR transducers but dissociates when misfolded proteins start to accumulate in the ER (Lee, 2005).

**Figure 2.**
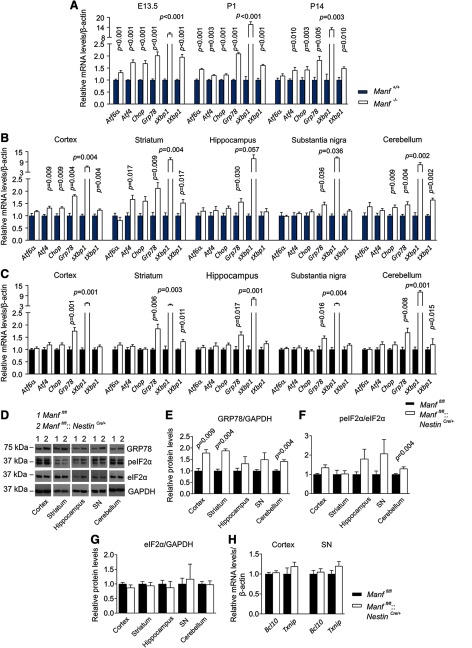
Upregulation of UPR genes in the brains of both conventional and conditional MANF KO mice. ***A***, qPCR analysis of UPR genes in the brains of E13.5 embryos and P1 and P14 pups of *Manf^-/-^* and *Manf^+/+^* mice (*n *=* *5–9). ***B***, qPCR analysis of UPR markers in specific brain regions of five-week-old *Manf^-/-^* and *Manf^+/+^* female mice (*n = *3–6). ***C***, qPCR analysis detecting changes of UPR markers in different brain parts of two-month-old *Manf^fl/fl^::Nestin^Cre/+^* female mice and *Manf^fl/fl^* female littermates (*n *=* *4–9). ***D***, Representative Western blottings of brain homogenates comparing protein levels of GRP78, p-eIF2α, eIF2α, and GAPDH between *Manf^fl/fl^::Nestin^Cre/+^* and *Manf^fl/fl^* male mice. Optical density-based quantitation of protein levels in immunoblots, where GRP78 is normalized to GAPDH (***E***), p-eIF2α to eIF2α (***F***), and eIF2α to GAPDH (***G***; *n = *4–6). ***H***, mRNA levels of *Bcl10* and *Txnip* in the cortex and SN of two-month-old *Manf^fl/fl^::Nestin^Cre/+^* and *Manf^fl/fl^* female mice (*n *=* *4–8). Results are scaled to the average value of the control samples. Mann–Whitney *U* test or Student’s *t* test was used for statistical analysis. Data are expressed as mean ± SEM.

First, the qPCR analysis performed on cDNA from the brains of *Manf^+/+^* and *Manf^-/-^* mice indicated that the mRNA levels of all the UPR-related genes from three UPR pathways, *Atf4*, *Atf6*α, C/EBP homologous protein (*Chop*), *Grp78*, *sXbp1* (activated by IRE1α endoribonuclease activity), and total *Xbp1* (*tXbp1*), were upregulated in the embryonic E13.5 *Manf^-/-^* mice brains and postnatally in the brains from newborn and two-week-old *Manf^-/-^* mice (Fig. 2*A*; Table 1). Next, the UPR induction was analyzed in the brain tissue of five-week-old *Manf^-/-^* mice at the age of manifestation of high blood glucose levels and diabetes (Lindahl et al., 2014). The brain regions analyzed were the cortex, striatum, hippocampus, SN and cerebellum. The *Atf6*α mRNA levels were not increased in any brain region analyzed from five-week-old *Manf^-/-^* mice (Fig. 2*B*; Table 1). However, mRNAs encoding for *Chop* and *Atf4*, which are synthesized and activated despite the translational initiation block in the PERK pathway, were both upregulated in the cortex of *Manf^-/-^* mice (Fig. 2*B*; Table 1). Interestingly, mRNA levels of the spliced form of a transcription factor XBP1 (*sXbp1*) were significantly upregulated by 6.1- to 11.5-fold in the cortex, striatum, SN, and cerebellum, indicating the activation of the IRE1α signaling pathway (Fig. 2*B*; Table 1). In addition to *sXbp1*, the mRNA levels of *Grp78* were significantly increased in all examined brain areas of five-week-old *Manf^-/-^* mice.

As experimentally-induced hyperglycemia and high-fat diet induced diabetes have been shown to increase ER stress and UPR activation in rodent brains (Zhao et al., 2015; Sims-Robinson et al., 2016), we studied the effect of MANF removal devoid of the hyperglycaemic effect in the brain tissue of *Manf^fl/fl^::Nestin^Cre/+^* mice. We examined UPR-related gene expression in the same brain regions of two-month-old *Manf^fl/fl^::Nestin^Cre/+^* mice and their *Manf^fl/fl^* littermates. The increase in *Xbp1* splicing in *Manf^fl/fl^::Nestin^Cre/+^* mice varied between brain regions, there was a 4.7-fold increase in the cortex, 3.7-fold increase in the striatum, 7.1-fold increase in the hippocampus, 4.4-fold increase in the SN, and as much as an 11.2-fold increase in the cerebellum (Fig. 2*C*; Table 1). In addition, mRNA levels of *Grp78* were significantly increased in all brain regions studied. Thus, upregulation of UPR genes *Grp78* and *sXbp1* was shared between conventional *Manf^-/^* ^-^ mice and conditional *Manf^fl/fl^::Nestin^Cre/+^* mice, confirming that this is a phenotype caused by MANF deficiency and not diabetes.

Next, we estimated protein levels of two ER stress markers, GRP78 and phosphorylated eukaryotic translation initiation factor 2α (p-eIF2α), in the brain homogenates of *Manf^fl/fl^* and *Manf^fl/fl^::Nestin^Cre/+^* mice. During ER stress, activation of PERK results in phosphorylation of eIF2α, leading to global translational repression (Walter and Ron, 2011). Results showed that the protein levels of GRP78 were significantly increased in the cortex, striatum and cerebellum (Fig. 2*D*,*E*; Table 1). Furthermore, the phosphorylation of eIF2α was significantly increased in the cerebellum, suggesting activation of the PERK pathway as well (Fig. 2*D*,*F*), but the expression of eIF2α was not increased (Fig. 2*D*,*G*). Despite the increase in the phosphorylation of eIF2α, the mRNA levels of *Atf4* were not upregulated in conditional *Manf^fl/fl^::Nestin^Cre/+^* brain.

In addition to its endoribonuclease (RNase) activity, IRE1α has a kinase activity that in ER stress, is responsible for recruitment of TRAF2 and further activation of the pro-inflammatory pathway, the nuclear factor κ subunit (NF-κB; Walter and Ron, 2011; Tam et al., 2012). Recently, it was shown that exogenously added MANF reduces activation NF-κB pathway and downregulates B-cell lymphoma 10 (*BCL10*) mRNA expression in cytokine-treated human pancreatic islets *in vitro* (Hakonen et al., 2018). BCL10 is a pro-apoptotic protein and an upstream regulator of the NF-κB pathway (Ruland et al., 2001). Silencing of MANF in a cytokine-treated human β cell line results in an increase of *BCL10* mRNA expression (Hakonen et al., 2018). Therefore, we also analyzed the mRNA levels of *Bcl10* in *Manf^fl/fl^::Nestin^Cre/+^* mice. However, we did not see changes in the expression levels of *Bcl10* mRNA in the cortex and SN of *Manf^fl/fl^::Nestin^Cre/+^* mice compared with *Manf^fl/fl^* mice (Fig. 2*H*; Table 1).

Activation of IRE1α RNase activity has been shown to induce expression of thioredoxin interacting protein (Txnip) by degradation of *Txnip* mRNA destabilizing miRNA-17 (Lerner et al., 2012). TXNIP expression has been associated with guiding β cells to terminal UPR following IRE1α hyperphosphorylation, oligomerization and increased inflammatory signaling (Morita et al., 2017). Therefore, we measured the mRNA levels of *Txnip*, but results showed no changes in the levels of *Txnip* mRNA expression in the cortex or SN of two-month-old *Manf^fl/fl^::Nestin^Cre/+^* mice (Fig. 2*H*; Table 1).

### All three UPR pathways are activated in MANF-deficient mouse brain during aging

**Figure 3.**
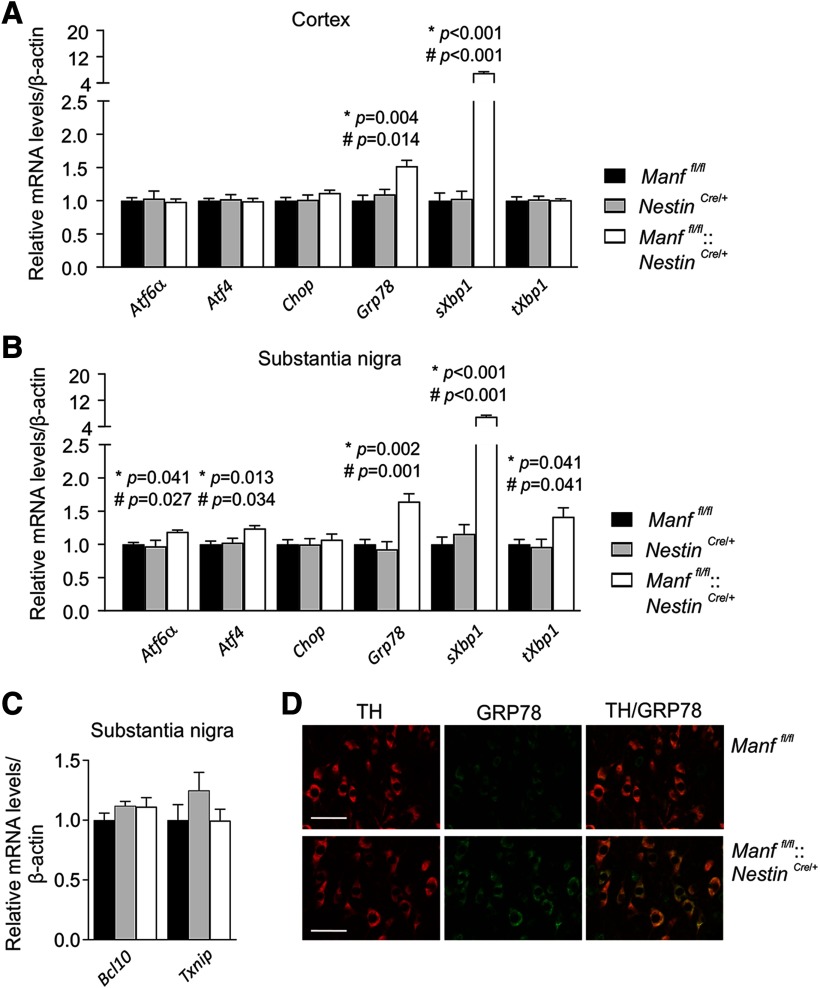
UPR remains activated in the brains of conditional MANF KO mice during aging. ***A***, qPCR analysis of UPR gene expression in the cortex samples of 16-month-old *Manf^fl/fl^::Nestin^Cre/+^*, *Manf^fl/fl^*, and *Nestin^Cre/+^* male mice (*n *=* *5–6). ***B***, Similar analysis of the SN from 16-month-old male mice (*n *=* *4–5). ***C***, mRNA levels of *Bcl10* and *Txnip* measured from the SN of 16-month-old *Manf^fl/fl^::Nestin^Cre/+^*, *Manf^fl/fl^*, and *Nestin^Cre/+^* male mice (*n *=* *4–6). ***D***, Co-localization of TH and GRP78 in the SN of *Manf^fl/fl^::Nestin^Cre/+^* and *Manf^fl/fl^* female mice. Scale bar: 50 μm. Results are scaled to the average value of the control samples. One-way ANOVA followed by Tukey’s *post hoc* test was used for statistical analysis. Data are expressed as mean ± SEM; *p Manf^fl/fl^ versus *Manf^fl/fl^::Nestin^Cre/+^*, and #*p Nestin^Cre/+^* versus *Manf^fl/fl^::Nestin^Cre/+^*.

To examine whether UPR activation continues during aging, we analyzed UPR genes in the cortex and SN of 16-month-old *Manf^fl/fl^::Nestin^Cre/+^* mice. Similarly to the two-month-old mice, both *Grp78* and *sXbp1* mRNA levels were almost identically upregulated in the cortex of 16-month-old mice (Fig. 3*A*; Table 1). This time, also *Nestin^Cre/+^* mice were included as another control, but the results showed similar mRNA levels between *Manf^fl/fl^* and *Nestin^Cre/+^* mice. Interestingly, in the SN of 16-month-old *Manf^fl/fl^::Nestin^Cre/+^* mice, expression of *Atf6*α and *Atf4* was also upregulated when compared with control mice, indicating activation of the ATF6 and PERK pathways in addition to the IRE1α pathway (Fig. 3*B*; Table 1). In comparison with the situation in younger mice, when only the IRE1α pathway was activated, 16-month-old *Manf^fl/fl^::Nestin^Cre/+^* mice had all three UPR pathways activated in the SN.

We also analyzed mRNA levels of both *Bcl10* and *Txnip* in the SN of 16-month-old *Manf^fl/^* ^fl^, Nestin^Cre^*^/+^* and *Manf^fl/fl^::Nestin^Cre/+^* mice. These markers, however, had still not changed (Fig. 3*C*; Table 1). Finally, GRP78 expression was confirmed to localize to TH-positive dopamine neurons in the SN of *Manf^fl/fl^* and *Manf^fl/fl^::Nestin^Cre/+^* mice by immunohistochemical costaining of GRP78 and TH (Fig. 3*D*).

### MANF deficiency does not affect the number of midbrain dopamine neurons during ageing

**Figure 4.**
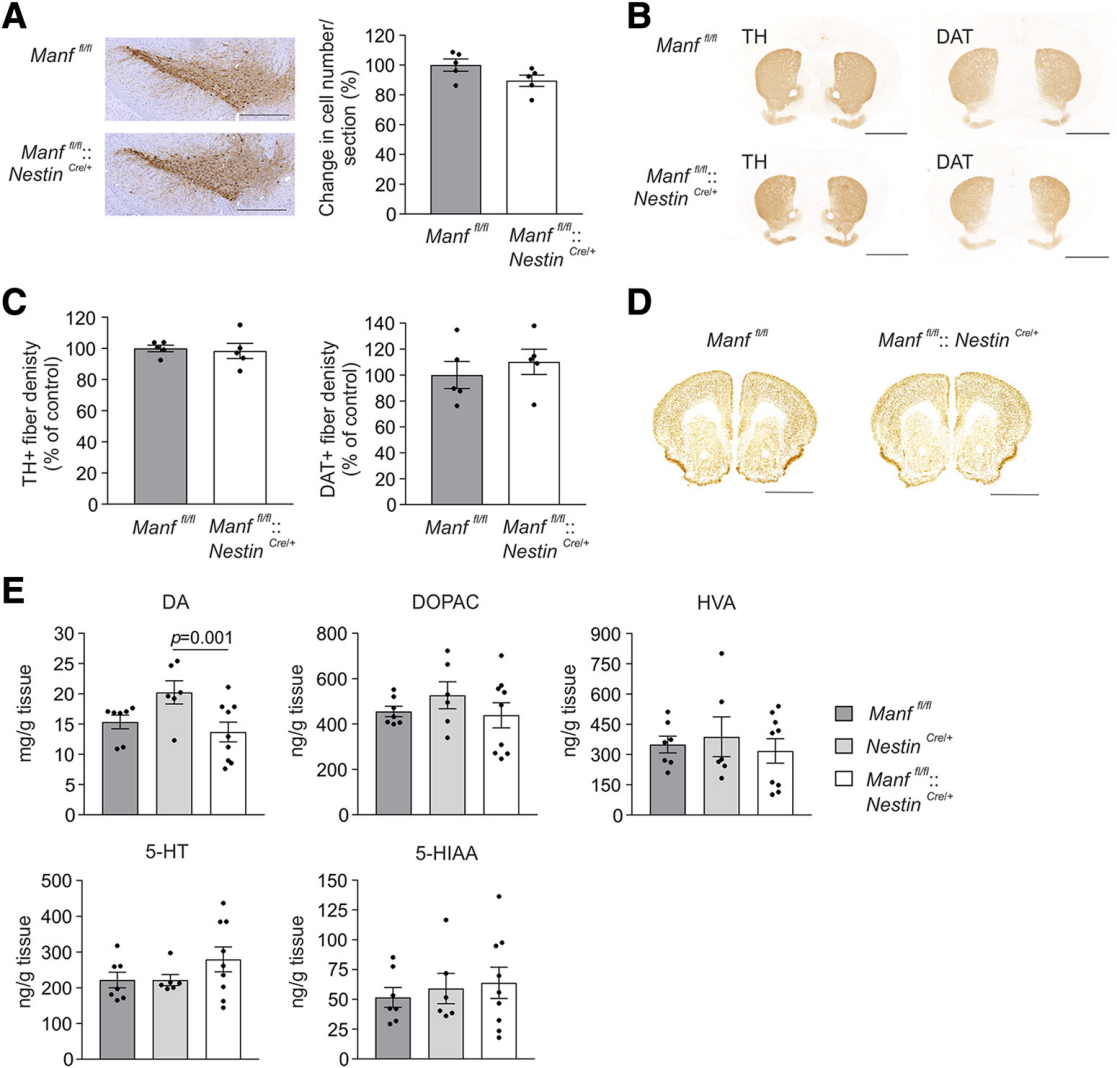
Loss of MANF in the brain does not cause degeneration of nigrostriatal dopamine neurons *in vivo*. ***A***, Representative pictures of TH-stained coronal sections of the SNpc and quantification of dopamine neurons per section of the SNpc from one-year-old *Manf^fl/fl^* and *Manf^fl/fl^::Nestin^Cre/+^* female mice (*n *=* *5). Scale bar: 500 μm. ***B***, Representative figures of TH-immunoreactivity and DAT-immunoreactivity in coronal striatal sections. Scale bar: 2 mm. ***C***, Measurement of optical density of TH-positive and DAT-positive dopamine fibers in one-year-old *Manf^fl/fl^::Nestin^Cre/+^* female mice and their *Manf^fl/fl^* littermate controls (*n = *5). ***D***, Representative NeuN-immunostaining in the sections from one-year-old *Manf^fl/fl^* and *Manf^fl/fl^::Nestin^Cre/+^* female mice. Scale bar: 2 mm. ***E***, HPLC analysis of monoamine metabolites measured from striatal samples of 16-month-old male mice (*n *=* *6–8). DA, dopamine; DOPAC, 3,4-dihydroxyphenylacetic acid; HVA, homovanillic acid; 5-HIAA, 5-hydroxyindoleacetic acid; 5-HT, 5-hydroxytryptamine. For statistical analysis, the Student’s *t* test and Kruskal–Wallis test followed by Mann–Whitney *U* test were used. Data are presented as mean ± SEM. See Extended Data Figure 4–1 for the further measurement of TH and DAT mRNA and protein levels in the striatum and SN.

Previous studies have implied that chronic UPR activation has an impact on neuronal survival in the brain as ER stress is associated with neurodegeneration (Hetz and Saxena, 2017; Lindholm et al., 2017). Thus, the chronic ER stress found in the brains of *Manf^fl/fl^::Nestin^Cre/+^* mice would indicate decreased survival, degeneration, and loss of neurons in the brains of these mice. Furthermore, MANF has been shown to be expressed in a portion of the TH-positive neurons in the SNpc (Lindholm et al., 2008) and therefore could affect their maintenance. To investigate the role of MANF as a physiological trophic factor for midbrain dopamine neurons, we analyzed the number of surviving nigrostriatal dopamine neurons of mice lacking endogenous MANF in the brain. Dopamine neurons in the SNpc were visualized by immunoreactivity to TH, a rate-limiting enzyme in dopamine synthesis. Relative quantification of TH-stained neurons in the SNpc showed no loss of dopamine neurons in one-year-old *Manf^fl/fl^::Nestin^Cre/+^* mice (Fig. 4*A*; Table 1). The axonal branches and striatal terminals of nigral neurons were detected by immunostaining of striatal sections with antibodies detecting TH and dopamine transporter (DAT; Fig. 4*B*). Optical densities of TH-positive and DAT-positive staining in the dorsal striatum were not altered, indicating no detectable changes in the striatal innervation of nigral dopamine neurons in one-year-old *Manf^fl/fl^::Nestin^Cre/+^* mice (Fig. 4*C*; Table 1). Moreover, we stained coronal brain sections including cortex and striatum with a neuronal NeuN antibody, but no obvious changes in the structure or number of positive cells were observed between one-year-old *Manf^fl/fl^::Nestin^Cre/+^* and *Manf^fl/fl^* mice (Fig. 4*D*).

In addition to morphology, striatal monoamine levels were measured to examine possible changes in the activity of dopamine neurons. The levels of dopamine and its metabolites, DOPAC and HVA, were measured from the striatal samples of 16-month-old mice by HPLC, i.e., at the time when all three UPR pathways are activated in MANF-deficient mice. The results showed no differences in the production of dopamine or its metabolites between *Manf^fl/fl^* and *Manf^fl/fl^::Nestin^Cre/+^* mice (Fig. 4*E*; Table 1). Unexpectedly, *Nestin^Cre/+^* mice had significantly elevated dopamine levels when compared with *Manf^fl/fl^::Nestin^Cre/+^* mice, but there were no differences in the levels of DOPAC or HVA. Moreover, the levels of serotonin (5-hydroxytryptamine) and its metabolite 5-hydroxyindoleacetic acid were measured, and as a result, no differences were observed between the groups (Fig. 4*E*; Table 1).

To assess possible changes in TH and DAT levels, we studied their expression at mRNA and protein levels. The mRNA levels of *Th* and *Dat* in the striatum and levels of *Th* in the SN were analyzed by qPCR and the results showed no differences in the level of their expression between two-month-old *Manf^fl/fl^* and *Manf^fl/fl^::Nestin^Cre/+^* mice (Extended Data Fig. 4-1*A*; Extended Data Table 1-1). Additionally, Western blot analysis indicated no changes in the protein levels of TH and DAT in the striatum or TH protein levels in the SN in *Manf^fl/fl^::Nestin^Cre/+^* mice compared with control *Manf^fl/fl^* mice (Extended Data Fig. 4-1*B*,*C*; Extended Data Table 1-1). Thus, MANF deficiency does not affect the expression of TH or DAT in the midbrain of young adult mice.

### Mice lacking MANF in the brain exhibit normal motor behavior

To investigate the effect of MANF absence from the nervous system on functional level, we characterized behavior of *Manf^fl/fl^::Nestin^Cre/+^* mice using a battery of different behavioral tests. The nigrostriatal pathway controls the initiation of movements and the cerebellum is important for the coordination of movements. Therefore, we analyzed motor behavior of MANF-deficient mice. Although the *Nestin^Cre/+^* line has been reported to have unaltered motor behavior, learning and memory, we still used this mouse line as a control, with *Manf^fl/fl^* littermate controls for *Manf^fl/fl^::Nestin^Cre/+^* mice (Giusti et al., 2014). Mice were tested at the age of four months and most tests were repeated in the same mice at the age of one year.

**Figure 5.**
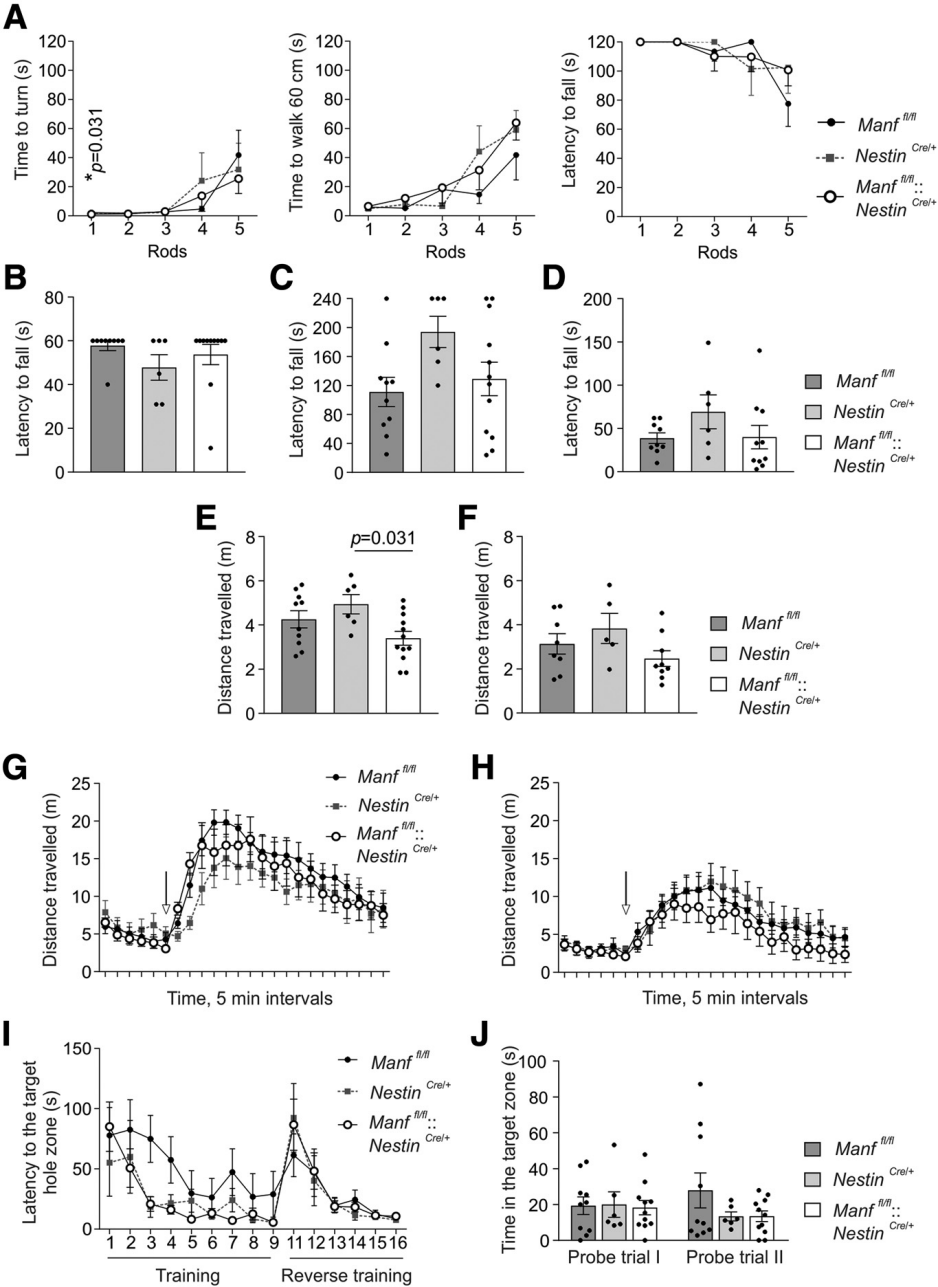
Lack of MANF causes no alterations in motor behavior, response to amphetamine, or learning and memory. ***A***, Multiple static rod test showing time to turn, time to walk 60 cm, and latency to fall using rods of different thickness from thicker (1) to thinner (5) in four-month-old *Manf^fl/fl^* (*n *=* *10), *Nestin^Cre/+^* (*n *=* *6), and *Manf^fl/fl^::Nestin^Cre/+^* (*n *=* *11) mice. ***B***, In the wire hanger test, latency to fall was measured in four-month-old *Manf^fl/fl^* (*n *=* *10), *Nestin^Cre/+^* (*n *=* *6), and *Manf^fl/fl^::Nestin^Cre/+^* (*n *=* *11) mice. Latency to fall in an accelerating rotarod test in four-month-old *Manf^fl/fl^* (*n *=* *10), *Nestin^Cre/+^* (*n *=* *6), and *Manf^fl/fl^::Nestin^Cre/+^* (*n *=* *12) mice (***C***) and in one-year-old *Manf^fl/fl^* (*n *=* *9), *Nestin^Cre/+^* (*n *=* *6), and *Manf^fl/fl^::Nestin^Cre/+^* (*n *=* *10) mice (***D***). Spontaneous locomotor activity in an open-field arena monitored for 30 min in four-month-old *Manf^fl/fl^* (*n *=* *10), *Nestin^Cre/+^* (*n *=* *6), and *Manf^fl/fl^::Nestin^Cre/+^* (*n *=* *12) mice (***E***) and one-year-old *Manf^fl/fl^* (*n *=* *8), *Nestin^Cre/+^* (*n *=* *5), and *Manf^fl/fl^::Nestin^Cre/+^* (*n *=* *9) mice (***F***). Hyperactivity response following amphetamine administration (3 mg/kg, i.p.) after habituation in four-month-old *Manf^fl/fl^* (*n *=* *10), *Nestin^Cre/+^* (*n *=* *6), and *Manf^fl/fl^::Nestin^Cre/+^* (*n *=* *11) mice (***G***) and in 14-month-old *Manf^fl/fl^* (*n *=* *9), *Nestin^Cre/+^* (*n *=* *6), and *Manf^fl/fl^::Nestin^Cre/+^* (*n *=* *9) mice (***H***). The arrow points out the time of amphetamine injection. ***I***, In the Barnes maze test, mice were subjected to nine training sessions prior to the first probe trial and six reverse training sessions before the second probe trial. Latency to find the target zone over training sessions is presented for four-month-old *Manf^fl/fl^* (*n *=* *10), *Nestin^Cre/+^* (*n *=* *6), and *Manf^fl/fl^::Nestin^Cre/+^* (*n *=* *11) mice. ***J***, Time spent around the target hole zone is presented for the first and second probe trials of the Barnes maze test. All the mice used in the behavioral test were male mice. For statistical analysis, Kruskal–Wallis test, one-way ANOVA, and two-way repeated measures ANOVA followed by Tukey’s *post hoc* test were used; *p Manf^fl/fl^ versus *Manf^fl/fl^::Nestin^Cre/+^*. Data are presented as mean ± SEM.

The performance in the multiple static rod test measuring balance was similar between four-month-old *Manf^fl/fl^*, *Manf^fl/fl^::Nestin^Cre/+^* and *Nestin^Cre/+^* mice (Fig. 5*A*; Table 1). In the wire hanger test, all mice had a similar latency to fall, suggesting unaltered motor strength (Fig. 5*B*; Table 1). Furthermore, the accelerated rotarod test measuring motor learning and coordination showed no alterations in the behavior of four-month-old (Fig. 5*C*; Table 1) or 12-month-old *Manf^fl/fl^::Nestin^Cre/+^* (Fig. 5*D*; Table 1) mice. To study the general locomotor activity, mice were placed in an open field and their activity monitored for 30 min. As Figure 5*E* illustrates, four-month-old *Manf^fl/fl^::Nestin^Cre/+^* mice moved less than *Nestin^Cre/+^* mice, but there was no difference in activity compared with *Manf^fl/fl^* littermates (Table 1). Furthermore, there were no changes in the locomotor activity between 12-month-old mice either (Fig. 5*F*; Table 1).

The functionality of the dopamine system in *Manf^fl/fl^::Nestin^Cre/+^* mice was analyzed by their responses to a dopaminergic stimulant. Amphetamine releases dopamine from vesicles in dopamine neurons and elevates striatal extracellular dopamine levels causing hyperactivity in mice (Yates et al., 2007). The response to D-amphetamine measured by locomotor activity was similar between *Manf^fl/fl^::Nestin^Cre/+^* and control mice at the age of four months (Fig. 5*G*; Table 1) and 14 months (Fig. 5*H*; Table 1), although the response to amphetamine was in general more modest in older mice. These results suggest that both dopamine uptake and release in the MANF-deficient striatum are normal.

Long-term memory formation relies on the synthesis of new mRNA and proteins, but in the case of ER stress, and particularly PERK pathway activation, protein translation is attenuated (Ohno, 2018). Indeed, hippocampal ER stress has been demonstrated to cause memory dysfunction (Zhang et al., 2014). Since we observed increased UPR activation in *Manf^fl/fl^::Nestin^Cre/+^* mice, we decided to investigate their spatial long-term memory by using the Barnes maze test, which consists of training trials and probe test trials measuring learning and long-term memory, respectively. In the second part of the test, the mice are trained to find the new location of the escape box to test behavioral flexibility. The results showed that four-month-old mice had a similar learning pattern independent of the genotype (Fig. 5*I*; Table 1) and we could not see differences between mice at one year of age either, indicating no alteration in learning (data not shown). In the memory test, the time spent in the target hole zone represents spatial long-term memory. The first and second probe trials for four-month-old mice showed that *Manf^fl/fl^*, *Manf^fl/fl^::Nestin^Cre/+^*, and *Nestin^Cre/+^* mice spent a similar amount of time in the target hole zone (Fig. 5*J*; Table 1) and similar behavior was observed for one-year-old mice (data not shown). Furthermore, there were no differences between genotype groups in any other of the parameters tested, which were latency to find the target hole zone, number of visits in the target hole zone, and errors made finding the target hole zone (data not shown). Thus, we found no defects in learning and long-term memory of *Manf^fl/fl^::Nestin^Cre/+^* mice.

### Cultured cortical neurons from embryonic MANF KO mice show increased ER stress and vulnerability to chemically-induced ER stress

**Figure 6.**
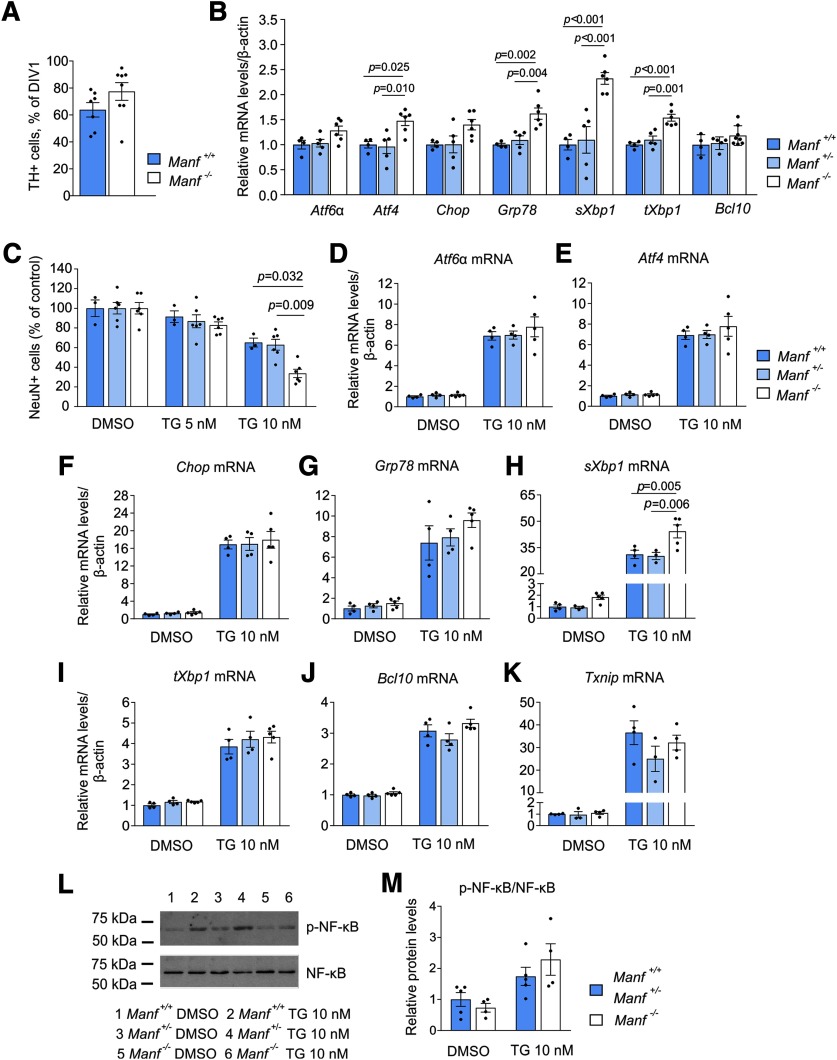
ER stress in embryonic cortical cultures of *Manf* ^-/-^ mice and their vulnerability to thapsigargin (TG). ***A***, Survival of embryonic midbrain dopamine neurons *in vitro* isolated from *Manf^+/+^* and *Manf^-/-^* mice (*n *=* *7–8). ***B***, Expression of UPR related and *Bcl10* genes in *Manf^+/+^*, *Manf^+/-^*, and *Manf^-/-^* cortical neurons cultured for 14 d and studied by qPCR (*n *=* *4–8). ***C***, Effect of the TG treatment on neuronal survival. Cortical neurons were treated with TG at DIV9, fixed after 48 h, and stained with the NeuN antibody (*n *=* *3–5). The loss of cells was quantified by comparing the neuron number with vehicle-treated cultures. Neurons were similarly treated with TG at DIV9, collected after 48 h and analyzed by qPCR for the expression of *Aft6α* (***D***), *Atf4* (***E***), *Chop* (***F***), *Grp78* (***G***), *sXbp1* (***H***), *tXbp1* (***I***), *Bcl10* (***J***), and *Txnip* (***K***) expression (*n *=* *3–5). ***L***, Western blottings presenting phosphorylated NF-κB and NF-κB expression in *Manf^+/+^*, *Manf^+/-^*, and *Manf^-/-^* cortical cultures after 48-h-long exposure to TG. ***M***, Quantification of Western blottings, where p-NF-κB is normalized to NF-κB (*n *=* *4–5). Results are scaled to the average value of the control samples. Each data point represents one animal and its value is an average of at least two wells. Data are expressed as mean ± SEM of at least three independent experiments. Experimental groups were compared by the Student’s *t* test, one-way ANOVA, or two-way ANOVA followed by Tukey’s multiple comparison test.

To study the survival of MANF KO neurons *in vitro*, we isolated dopamine neurons from the midbrain floor of E13.5 *Manf^+/+^* and *Manf^-/-^* embryos and cultured them for 5 d. The number of surviving TH-positive cells was measured by comparing the initial number of cells on DIV1 with the number of cells on day 5. The results showed no changes in the survival of non-treated TH-positive neurons from MANF KO mice compared with wild-type controls *in vitro* (Fig. 6*A*; Table 1).

Because of the scarce material obtained from a midbrain floor of one embryonic mouse, we could not plan controlled chemical treatments and biochemical analysis for MANF KO dopamine neurons. Therefore, we continued to study UPR activation *in vitro* using primary cortical neurons isolated from *Manf^+/+^*, *Manf^+/-^*, and *Manf^-/-^* embryos at day E16 or E17 as they can be easily obtained with large quantities from embryonic mice. First, we evaluated the ER stress levels of cortical neurons cultured for two weeks. The cultures consisted mostly of a heterogenic population of cortical neurons as they were treated with mitotic inhibitors to reduce non-neuronal cell proliferation. Increased UPR activation was found in *Manf^-/-^* cortical neurons when compared with neurons from both *Manf^+/+^* and *Manf^+/-^* embryos. Similarly to increased expression of UPR genes in the brain of *Manf^-/-^* embryos *in vivo* (Fig. 2*A*), expression of *Grp78*, *sXbp1*, and *tXbp1*, but not *Chop* and *Atf6*α, was increased in *Manf^-/-^* neurons cultured for 14 d *in vitro* (Fig. 6*B*; Table 1). In addition, the mRNA levels of *Atf4* were increased, suggesting activation of the PERK pathway. The *Bcl10* mRNA levels did not differ between genotypes (Fig. 6*B*; Table 1).

The vulnerability of *Manf^-/-^* cortical neurons was assessed by applying a chemical ER stressor, thapsigargin, to the cultures. Thapsigargin blocks the sarcoendoplasmic reticulum calcium transport ATPase (SERCA) pump and depletes Ca^2+^ stores from the ER, leading to UPR activation and cell death in various cell types, including neurons (Li and Hu, 2015). Thapsigargin at a concentration of 10 nM for 48 h led to a 66.3 ± 10.4% reduction in *Manf^-/-^* neuronal survival compared with dimethyl sulfoxide (DMSO)-treated neurons, whereas the decrease in survival of control *Manf^+/+^* neurons was 34.9 ± 7.9% and *Manf^+/-^* neurons 37.1 ± 13.4% (genotype–treatment interaction, *p = *0.039; Fig. 6*C*; Table 1). At 5 nM thapsigargin, no changes in the number of surviving neurons were detected (Fig. 6*C*). As a result, neurons without endogenous MANF were more vulnerable to thapsigargin-induced ER stress *in vitro.*

To examine further the effect of thapsigargin on neuronal cultures, the expression of several UPR markers was analyzed after a 48-h-long exposure to 10 nM thapsigargin. Upregulation of many UPR genes, *Atf6α*, *Atf4*, *Chop*, *Grp78*, *sXbp1*, and *tXbp1*, was detected after thapsigargin treatment in all genotypes (Fig. 6*D–I*; Table 1). Furthermore, expression of *Bcl10* was higher in thapsigargin-treated neurons compared with DMSO-treated ones (Fig. 6*J*; Table 1). The treatment with 10 nM thapsigargin resulted in a significant genotype–treatment interaction only regarding mRNA levels of *sXbp1* (*p *=* *0.012; Table 1). Thus, the results showed higher upregulation of *sXbp1* in *Manf^-/-^* neurons compared with *Manf^+/+^* and *Manf^+/-^* neurons (Fig. 6*H*). We also measured the mRNA levels of *Txnip* in the samples, and the levels were upregulated in response to thapsigargin but did not differ between the genotypes (Fig. 6*K*; Table 1).

Finally, as sustained ER stress is known to induce NF-κB activation by IRE1α activation (Kaneko et al., 2003), we also evaluated the phosphorylation of NF-κB by Western blotting (Fig. 6*L*). Because of a small sample size, we combined *Manf^+/+^* and *Manf^+/-^* samples together. Quantification of intensity in Western blotting bands showed only a trend towards increased phosphorylation of NF-κB in *Manf^-/-^* neurons after 10 nM thapsigargin treatment compared with that in control neurons (Fig. 6*M*; Table 1).

## Discussion

In this study, we elucidate the previously unknown function of endogenous MANF in the mouse brain. Previous studies have suggested a function for MANF in the maintenance of neurons based on neuroprotective and neurorestorative effects of MANF in different rodent degenerative disease models (Airavaara et al., 2009; Voutilainen et al., 2009; Yang et al., 2014; Neves et al., 2016). However, our results indicate for the first time that endogenous MANF is not needed for the maintenance of midbrain dopamine neurons in mice. It has been implicated, that accumulation of misfolded or aggregated proteins followed by severe chronic UPR causes neuronal death in neurodegenerative diseases. This study, however, questions this present view and shows that sustained but moderate UPR in the rodent brain is not detrimental.

The phenotype of conventional *Manf^-/-^* is dominated by the development of insulin-dependent diabetes, a severe growth defect and premature death (Lindahl et al., 2014). On the contrary, *Manf^fl/fl^::Nestin^Cre/+^* mice are viable and have a normal lifespan without diabetes or gross defects, but adult *Manf^fl/fl^::Nestin^Cre/+^* mice have a decreased body weight when compared with littermate *Manf^fl/fl^* controls. In a recent study, *Manf* knock-down by siRNA from the hypothalamus resulted in hypophagia and reduced body weight (Yang et al., 2017), suggesting that *Manf^fl/fl^::Nestin^Cre/+^* mice could be smaller due to hypophagia. However, *Nestin^Cre/+^* mice themselves have been reported with a growth defect due to reduced levels of mouse growth hormone (Galichet et al., 2010; Declercq et al., 2015). Therefore, the reduced weight in *Manf^fl/fl^::Nestin^Cre/+^* mice is affected by the *Nestin^Cre/+^* transgene and the effect of hypothalamic MANF deficiency on food intake requires more investigations.

Upon ER stress, the IRE1α receptor is autophosphorylated and its RNase activity leads to degradation of mRNAs and unconventional splicing of *Xbp1* mRNA (Walter and Ron, 2011). Our study reveals increased activation of the IRE1α RNase pathway in the brains of both conventional *Manf^-/-^* mice and conditional *Manf^fl/fl^::Nestin^Cre/+^* mice. In young adult *Manf^-/-^* mice, UPR activation seems more severe with increased expression of *Chop* mRNA, which may be partially due to the manifestation of hyperglycemia as cerebral ER stress has been reported in diabetic mice (Zhao et al., 2015). Spliced XBP1 induces the expression of UPR target genes, such as *Grp78* (Hetz and Saxena, 2017). Indeed, *Grp78* expression is also increased in tissue samples where splicing of *Xbp1* is elevated. The increased phosphorylation of eIF2α in the brains of *Manf^fl/fl^::Nestin^Cre/+^* mice does not result in increased expression of its downstream components *Atf4* nor *Chop* indicating only minor PERK pathway activation at young age. However, in 16-month-old *Manf^fl/fl^::Nestin^Cre/+^* mice, expression of *Atf4* and *Atf6*α are increased suggesting activation of both PERK and ATF6 pathways in the brain at older age. Interestingly, the IRE1α RNase pathway is chronically stimulated through the lifespan of *Manf^fl/fl^::Nestin^Cre/+^* mice. Therefore, supporting previous findings in other tissues (Wang et al., 2018; Hartman et al., 2019), we conclude that also in neurons MANF regulates UPR mainly through the IRE1α pathway.

In rats, exogenously added and striatally overexpressed MANF protects dopamine neurons in the 6-hydroxydopamine model of Parkinson’s disease (Voutilainen et al., 2009; Hao et al., 2017). Here, we analyzed whether MANF deficiency and chronic UPR in the mouse brain leads to nigrostriatal neurodegeneration. It has been shown that in Parkinson’s disease dopamine neurons start to degenerate by losing their synapses and axons. This is observed both in patients and in Parkinson’s rodent disease models with clear Parkinson’s disease phenotype (Domanskyi et al., 2015). In this study, we measured the optical density of striatal dopamine fibers by two different markers, TH and DAT, and did not observe any differences in density between the genotypes. We also used even more sensitive measurements for dopamine neuronal degeneration by quantifying striatal dopamine concentrations and its metabolite levels with HPLC, and did not find any differences between the genotypes. The concentrations of the striatal samples were in range of 7.6–25.4 mg/g of tissue, and similar that we have published before (Albert et al., 2019). Based on these results, maintenance of dopamine neurons is not affected by the embryonic onset of MANF deficiency in the mouse brain, which supports another study showing MANF not being a crucial survival factor for dopamine neurons in *Caenorhabditis elegans* (Hartman et al., 2019).

Synaptic alteration and loss occur at early stages of neurodegenerative diseases, such as Alzheimer’s, Parkinson´s and Lewy body diseases (Hunn et al., 2015; Overk and Masliah, 2014). It has been demonstrated in mice that synapses can degenerate, although striatal dopamine fibers and dopamine levels stay unchanged (Galli et al., 2014). Therefore, in the future, it will be important to quantify the number of synapses (DeKosky and Scheff, 1990) and examine individual synapses of dopamine neurons in MANF-deficient mice using highly sensitive technologies, such as mass synaptometry (Gajera et al., 2019). Furthermore, changes in dopamine transmission at the synapses can also occur without dopamine neuron loss as shown in CDNF KO mice (Lindahl et al., 2020). Despite the unchanged response to amphetamine, detailed investigation of the dopamine neurotransmission in MANF-deficient mice would be justified in the future.

It is possible that embryonic deletion of MANF is compensated by factors that remain unknown. We confirmed that at least in the adult brain, loss of MANF is not compensated by its protein family member CDNF, but future studies on CDNF-MANF double KO mice are relevant. With possible functional compensation in mind, we also quantified the mRNA levels of *Bdnf*, *Th*, and *Dat* and protein levels of TH and DAT but found no changes in their levels between the genotypes. In future studies it will be important to eliminate embryonic compensatory effects in the MANF KO mice by deleting MANF specifically from the brain in adult mice. It has been shown that in ischemic brains, MANF is expressed in activated microglia (Shen et al., 2012). Other studies also demonstrate expression of MANF by myeloid cells (Neves et al., 2016; Mätlik et al., 2018). It is possible that MANF is expressed by microglia in our *Manf^fl/fl^::Nestin^Cre^* ^/+^ mice. However, we did not find detectable levels of *Manf* mRNA expression in the cortex of 16-month-old *Manf^fl/fl^::Nestin^Cre^* ^/+^ mice suggesting no microglial upregulation of MANF.

Normal locomotor activity of *Manf^fl/fl^::Nestin^Cre/+^* mice is in line with unaltered open field activity of *Manf^-/-^* mice (Lindahl et al., 2014). In *Nestin^Cre/+^* mice, slightly higher locomotor activity could be explained by their surprisingly higher striatal dopamine levels. However, this phenomenon might be due to differences in a genetic background as our breeding did not generate *Nestin^Cre/+^* littermate control mice alone. Although ER stress can impair long-term memory (Ohno, 2018), hippocampal sXBP1 overexpression, instead, has been shown to improve long-term memory through elevated *Bdnf* levels (Martínez et al., 2016). However, we did not observe changes in the hippocampal *Bdnf* mRNA levels in *Manf^fl/fl^::Nestin^Cre/+^* mice possibly explaining the unchanged memory consolidation. Thus, despite increased expression of *Grp78* and *sXbp1* mRNA in the hippocampus of *Manf^fl/fl^::Nestin^Cre/+^* mice, behavioral tests showed that MANF deficiency does not result in memory impairment.

We have shown that exogenously administered MANF before cerebral ischemia protects cortical neurons in rats (Airavaara et al., 2009) and poststroke delivery of MANF induces functional recovery of rats after cerebral ischemia (Mätlik et al., 2018). Moreover, the infarction size in the MANF-deficient brain is larger, indicating that endogenous MANF has a protective effect on cortical neurons against ischemic injury (Mätlik et al., 2018). Therefore, we examined ER stress in the *Manf* ^-/-^ primary cortical neurons. Cortical MANF KO neurons show increased ER stress levels, confirming that the increased UPR activation is neuronal. We also report decreased survival of thapsigargin-treated MANF-depleted cortical neurons, suggesting increased vulnerability of *Manf^-/-^* cortical neurons *in vitro*. Neurons in cortical cultures were quantified based on their immunoreactivity to NeuN, although we acknowledge the issues related to its use in primary cultures. It has been criticized that also astrocytes can show immunoreactivity to NeuN *in vitro* (Darlington et al., 2008) and that the loss of NeuN expression does not necessarily correlate with cell death (Unal-Cevik et al., 2004). Here, we used it as a marker for the vulnerability of neurons to chemically-induced ER stress comparing with untreated neurons.

Previously, we reported impairments in the cortical development of *Manf^-/-^* brain, but the adult cortex is morphologically normal (Tseng et al., 2017). Our results here show increased UPR activation in the brain of *Manf^-/-^* mice during embryonic and postnatal development, but activation seems to gradually decrease after birth. UPR has been shown to be tightly regulated during neurodevelopment and to play a critical role during maturation of neurons and in neurodevelopmental diseases (Laguesse et al., 2015; Godin et al., 2016; Gladwyn-Ng et al., 2018). Interestingly, a patient with a homozygous missense mutation in the MANF gene likely leading to a null mutation was presented with microcephaly and diabetes (Yavarna et al., 2015). Thus, the impact of MANF in the development of the human brain might differ compared with mice.

Whereas chronic UPR activation kills β cells (Lindahl et al., 2014), neuronal loss is not observed in the midbrain of *Manf^fl/fl^::Nestin^Cre/+^* mice. One of the actors determining the switch between adaptive and terminal UPR followed by IRE1α activation is TXNIP (Anthony and Wek, 2012). However, in *Manf^fl/fl^::Nestin^Cre/+^* mice midbrain the IRE1α pathway is activated without increased expression of *Txnip* mRNA. One of the differences between β cells and dopamine neurons is that β cells produce and secrete insulin at a high rate, making them vulnerable to additional ER perturbation. Although chronic ER stress in β cells leads to increased activation of the NF-κB pathway (Danilova et al., 2019a), this pathway is not upregulated in neuronal tissue lacking MANF. As expression of *Bcl10* mRNA or phosphorylation of NF-κB was not differently regulated between genotypes after thapsigargin administration, we suggest that the increased susceptibility of MANF KO neurons to thapsigargin is not related to the activation of the NF-κB pathway. We conclude that the IRE1α-TRAF2-NF-κB branch is not activated in MANF-deficient neurons.

A recent study suggests that MANF functions as a negative regulator of IRE1 and XBP1 activity in nematodes (Hartman et al., 2019). Our data demonstrating how ablation of *Manf* increases *sXbp1* levels agrees with this hypothesis. The IRE1α–XBP1 pathway is regarded as an UPR branch, which can, differently from others, also enhance cell viability (Lin et al., 2009). Activation of this pathway has also demonstrated neuroprotection in different *in vivo* models of CNS diseases (Ni et al., 2018). In the MPTP-induced animal model of Parkinson’s disease, for example, expression of sXBP1 protects dopamine neurons (Sado et al., 2009). We can hypothesize that in our mouse model, neurons are adapted to constant, non-pathologic activation of the IRE1α pathway increasing the capability to survive from the terminal UPR. Developmental deletion of XBP1 in the nervous system also causes ER stress in the mouse brain, which protects dopamine neurons, whereas reduction of XBP1 levels at the adult stage results in neurodegeneration (Valdés et al., 2014). The authors speculate that developmental adaptation to ER stress would explain the protection of dopamine neurons (Valdés et al., 2014). A similar adaptation might occur in our *Manf^fl/fl^::Nestin^Cre/+^* mice, and therefore, adult knock-down of *Manf* expression may lead to a deleterious outcome.

In conclusion, our study describes a mouse model with prolonged UPR activation in the brain. We discovered a different effect of sustained ER stress between neurons and β cells. Whereas terminal UPR kills β cells in the *Manf^-^* ^/-^ pancreas, there is no neurodegeneration in the midbrain dopamine system of *Manf^fl/fl^::Nestin^Cre/+^* mice despite the upregulation of UPR genes. However, the lack of MANF decreases survival of cultured cortical neurons when exposed to chemically-induced ER stress. This is the first study analyzing the role of endogenous MANF in the nigrostriatal dopamine system with the conclusion that in our transgenic mouse model, MANF expression does not seem to be needed for the proper function of midbrain dopamine neurons in the aging brain.

10.1523/ENEURO.0477-19.2019.f4-1 Extended Data Figure 4-1 ***A***, qPCR analysis showing the mRNA levels of *Th* from the striatum (*n *=* *4–9) and *Th* and *Dat* from the SN of two-month-old *Manf^fl/fl^* and *Manf^fl/fl^::Nestin^Cre/+^* female mice (*n *=* *4–8). Results are scaled to the average value of the control samples. ***B***, Representative Western blotting pictures of TH and DAT levels in the striatum and TH levels in the SN of two-month-old *Manf^fl/fl^* and *Manf^fl/fl^::Nestin^Cre/+^* female mice. ***C***, Quantification of Western blot analysis of TH and DAT in the striatum (*n *=* *5–6) and TH levels in the SN (*n *=* *4–6). For statistical analysis, the Student’s *t* test was used. Data are presented as mean ± SEM. Download Figure 4-1, TIF file.

10.1523/ENEURO.0477-19.2019.t1-1 Extended Data Table 1-1 Statistical analysis for extended data. Download .

## Synthesis

Reviewing Editor: Julie Andersen, Buck Institute Decisions are customarily a result of the Reviewing Editor and the peer reviewers coming together and discussing their recommendations until a consensus is reached. When revisions are invited, a fact-based synthesis statement explaining their decision and outlining what is needed to prepare a revision will be listed below. The following reviewer(s) agreed to reveal their identity: NONE. Note: If this manuscript was transferred from JNeurosci and a decision was made to accept the manuscript without peer review, a brief statement to this effect will instead be what is listed below.

This transferred manuscript from the Journal of Neuroscience has now been re-viewed by eNeuro. The consensus opinion is that the current study furthers previous work by the authors on MANF, particularly inclusion of the constitutive KO. Several means were utilized to identify a specific brain phenotype following MANF deletion, albeit unsuccessfully. However these studies have allowed the authors to rule out several possible outcomes, which is in itself an advancement in the field and points towards the need for more specific and sensitive bioassays or use of a conditional KO allowing adult onset loss of function.

The authors have made logical attempts to address the issues raised in the previous JNS review, including discussion of previous published data from their lab or others, attesting to limitations of the current study design and how this may be improved in next study. Responses have however not been incorporated into the revised manuscript itself and should be included in the Discussion (or where appropriate) in order to enhance its relevance for eNeuro readership. These include:

(1) Additional compensatory mechanisms may be present in the constitutive MANF knockout mouse model which may be overcome by deleting MANF in adult mice.

(2) “It has been shown that in Parkinson's disease dopamine neurons start to degenerate by losing their axons. This is observed both in patients and in PD models with clear PD phenotype (Domanskyi et al., 2015, Hum Gene Ther.; Albert et al. 2017, Genes;,Albert et al., 2019, J Neurosci Res.). In this study, we measured the optical density of striatal dopamine fibers by two different markers, TH and DAT, and did not observe any differences in density between the genotypes (Fig. 4C,D). We also used even more sensitive and highly linear measurements of dopamine neuronal degeneration by quantifying striatal dopamine concentrations and its metabolite levels with HPLC with electrochemical detection, and did not find any differences between the genotypes (Fig. 4E). The concentrations of the striatal samples were in range of 7.6-25.4 mg/g of tissue, and similar that we have published before (Albert et al., 2018; J Neurosci Res.).”

(3) Synaptic changes may have not been captured in Nestin-Cre; Manffl/fl model which may require ore sensitive technologies such as mass cytometry/CyTOF of single synapses (Reference: 1) Synapse loss in frontal cortex biopsies in Alzheimer's disease: correlation with cognitive severity. 1990. Ann. Neurol. 27, 457-464. 2) Pathogenesis of synaptic degeneration in Alzheimer's disease and Lewy body disease. 2014. Biochem. Pharmacol. 88, 508-516. 3) Mass synaptometry: High-dimensional multi parametric assay for single synapses, 2019, Journal of Neuroscience Methods 312 73-8374 4) Deficient Wnt signalling triggers striatal synaptic degeneration and impaired motor behaviour in adult mice. 2014. Nat. Commun. 5, 499. The authors are encouraged to add and expand this in the Discussion as appropriate.
