# Supplementary figures and images for: MANF Ablation Causes Prolonged Activation of the UPR without Neurodegeneration in the Mouse Midbrain Dopamine System
Source: eNeuro. 2020 Feb 14;7(1):ENEURO.0477-19.2019. doi: 10.1523/ENEURO.0477-19.2019 (PMC7053174; doi:10.1523/ENEURO.0477-19.2019)

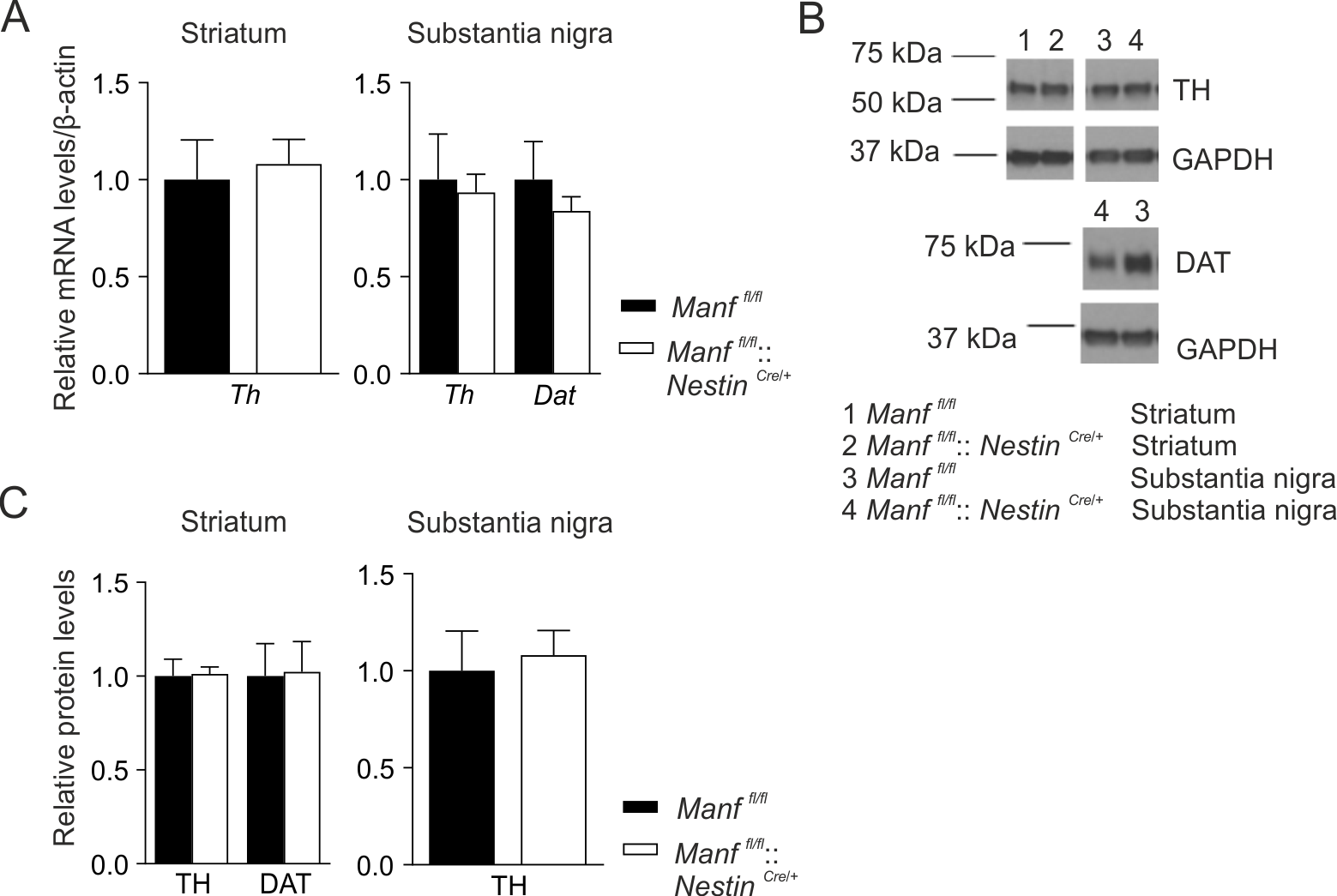

Supplement: Extended Data Figure 4-1 — A, qPCR analysis showing the mRNA levels of Th from the striatum (n = 4–9) and Th and Dat from the SN of two-month-old Manffl/fl and Manffl/fl::NestinCre/+ female mice (n = 4–8). Results are scaled to the average value of the control samples. B, Representative Western blotting pictures of TH and DAT levels in the striatum and TH levels in the SN of two-month-old Manffl/fl and Manffl/fl::NestinCre/+ female mice. C, Quantification of Western blot analysis of TH and DAT in the striatum (n = 5–6) and TH levels in the SN (n = 4–6). For statistical analysis, the Student’s t test was used. Data are presented as mean ± SEM. Download Figure 4-1, TIF file. [file enu-eN-NWR-0477-19-s01.tif]
