# Supplementary material for: MANF Ablation Causes Prolonged Activation of the UPR without Neurodegeneration in the Mouse Midbrain Dopamine System
Source: eNeuro. 2020 Feb 14;7(1):ENEURO.0477-19.2019. doi: 10.1523/ENEURO.0477-19.2019 (PMC7053174; doi:10.1523/ENEURO.0477-19.2019)
Supplement: Extended Data Table 1-1 — Statistical analysis for extended data. Download . [file enu-eN-NWR-0477-19-s02.docx]

**Table 1-1. Statistical analysis for extended data.**

| Location | Dataset | Data structure | Type of test | Power |
| --- | --- | --- | --- | --- |
| a | Fig. 4-1A | Normal distribution | Two-tailed *t* test | Striatum *Th* *t*(11)=0.34, *p*=0.738; SN *Th* *t*(10)=0.32, *p*=0.760; SN *Dat* *t*(10)=0.95, *p*0.363 |
| b | Fig. 4-1C | Normal distribution | Two-tailed *t* test | Striatum TH *t*(9)=0.11, *p*=0.914; Striatum DAT *t*(9)=0.096, *p*=0.925; SN TH *t*(8)=0.80, *p*=0.449 |
